# An *N-terminal acidic β-sheet domain* is responsible for the metal-accumulation properties of amyloid-β protofibrils: a molecular dynamics study

**DOI:** 10.1007/s00775-024-02061-1

**Published:** 2024-05-29

**Authors:** Carlos Z. Gómez-Castro, Liliana Quintanar, Alberto Vela

**Affiliations:** 1 Conahcyt—Universidad Autónoma del Estado de Hidalgo, Km 4.5 Carr. Pachuca-Tulancingo, Mineral de La Reforma, 42184 Hidalgo, Mexico; 2 Department of Chemistry, Cinvestav, Av. Instituto Politécnico Nacional 2508, CDMX, San Pedro Zacatenco, 07360 Gustavo A. Madero, Mexico

**Keywords:** Amyloid protofibrils, Molecular dynamics, Metal cations, Aggregation, Alzheimer’s disease, β-sheet

## Abstract

The influence of metal ions on the structure of amyloid-$\beta$ (Aβ) protofibril models was studied through molecular dynamics to explore the molecular mechanisms underlying metal-induced Aβ aggregation relevant in Alzheimer’s disease (AD). The models included 36-, 48-, and 188-mers of the Aβ_42_ sequence and two disease-modifying variants. Primary structural effects were observed at the N-terminal domain, as it became susceptible to the presence of cations. Specially when β-sheets predominate, this motif orients N-terminal acidic residues toward one single face of the β-sheet, resulting in the formation of an acidic region that attracts cations from the media and promotes the folding of the N-terminal region, with implications in amyloid aggregation. The molecular phenotype of the protofibril models based on Aβ variants shows that the AD-causative D7N mutation promotes the formation of N-terminal β-sheets and accumulates more Zn^2+^, in contrast to the non-amyloidogenic rodent sequence that hinders the β-sheets and is more selective for Na^+^ over Zn^2+^ cations. It is proposed that forming an acidic β-sheet domain and accumulating cations is a plausible molecular mechanism connecting the elevated affinity and concentration of metals in Aβ fibrils to their high content of β-sheet structure at the N-terminal sequence.

**Graphic abstract:**

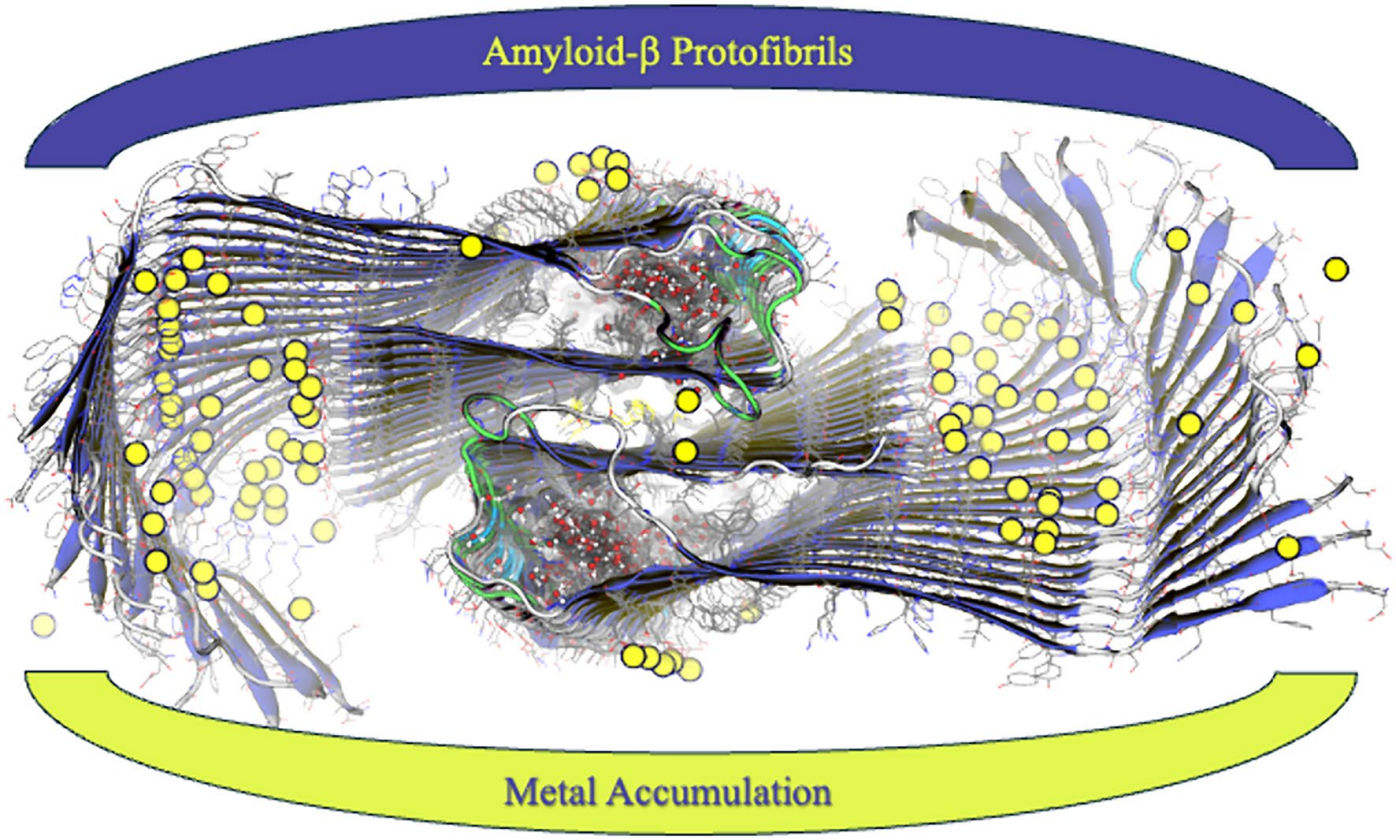

**Supplementary Information:**

The online version contains supplementary material available at 10.1007/s00775-024-02061-1.

## 1. Introduction

Considered by the World Health Organization (WHO) as a public health priority, Alzheimer’s Disease (AD) accounts for approximately 60–70% of dementia cases worldwide [1, 2]. This neurodegenerative disorder causes deterioration of memory and cognitive ability to perform everyday activities [1, 3]. One of the hallmarks of AD is the presence of fibrillar protein deposits in the brain known as senile or amyloid plaques composed mainly by the amyloid-β peptide (Aβ). It was originally conjectured that the accumulation of Aβ and its aggregation into amyloid fibrils would be causative in neurodegeneration leading to AD [4]. However, later findings point to oligomeric forms of Aβ as the most neurotoxic species [5, 6].

**Fig. 1:**
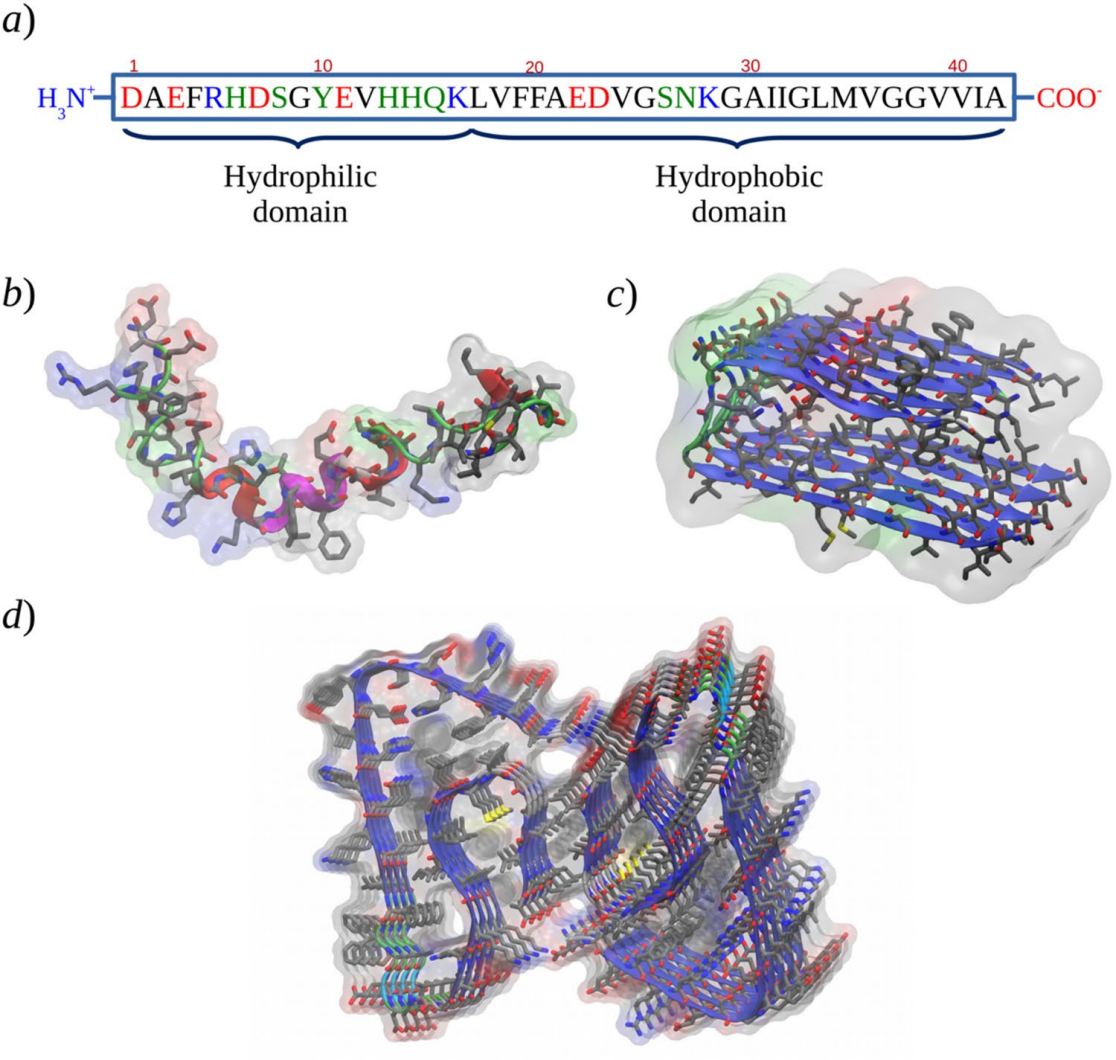
Structure of the Aβ protein. **a** Primary structure. **b** NMR structure in trifluoroethanol/water solution (PDB: 1z0q). **c** Solid-state NMR structure of the hydrophobic core of Aβ_42_ fibrils (PDB: 2beg). **d** Near-atomic resolution cryo-EM structure of Aβ_42_ fibrils (PDB: 5oqv). The amino acid type is highlighted in red for acidic, blue for basic, green for polar, and gray for non-polar residues

Aβ is produced from the proteolytic cleavage of the amyloid precursor protein (APP) by β- and γ-secretases, yielding Aβ peptides with 40 to 43 amino acid residues [7]. Among the different Aβ variants [8–10], Aβ_42_ is the most abundant species in AD amyloid plaques, it is the most aggregation-prone, and it produces toxic oligomers [10–12]. The peptide may be divided into two domains: the hydrophilic N-terminal (residues 1-16) that binds metal ions, such as copper or zinc; and the hydrophobic C-terminal domain (17-42), engaged in β-sheet formation (Fig. 1a) [13, 14]. Aβ displays structural flexibility and sensitivity to environmental factors such as temperature, concentration, pH, and interaction with metal ions or membranes. In non-polar solvents or membranes, Aβ adopts predominantly β structure, [15–17] whereas in aqueous solution it may be found with an extended random coil structure (Fig. 1b) [18], with a tendency to aggregate into oligomeric species. Soluble-Aβ oligomers can go from two and up to hundreds of Aβ peptides with unknown structure; however, increasing oligomer size (dimer, trimer, etc.) has been associated with higher contents of β structure with a concomitant increase in toxicity [19, 20]. Larger aggregates form non-crystalline and pleomorphic deposits of insoluble fibrils. Even though high-definition structural characterization of fibrils is difficult, several structural features have been described and are briefly discussed below [21–24].

The structure of amyloid fibrils contains the cross-β motif, consisting of β-sheets formed along the fibril, with peptide strands extending approximately perpendicular to the long fibril axis and orienting inter-strand backbone hydrogen bonds approximately parallel to the same direction [21–23]. This arrangement, also known as a filament, may be seen as an array or a layer of protein molecules aligned along the fibril axis (Fig. 1c). In Aβ fibrils, a minimum of two or three of these layers (with two- or three-fold symmetry) are commonly associated in forming a structural unit known as protofibril [25–29]. Lateral association and supercoiling of two or more protofibril subunits would form “mature” fibrils [23].

Each Aβ peptide in the cross-β motif has been proposed to fold into a β-sheet-turn-β-sheet motif [27, 30, 31], although many different folds have been described so far [32–42]. (Fig. 1d) This arrangement allows the formation of an “intra-layer” steric zipper, where side chains from two stacking β-sheets interdigit and confront complementary hydrophobic surfaces [43, 44]. Steric zippers formed in the interface between two Aβ layers are also possible [28], and provide structural stability to the hydrophobic core of protofibrils. Amyloid fibrils are generally observed as twisted structures within a range of crossover lengths and fibril widths [45], although non-twisted topologies have been observed for amyloid fibrils seeded from brain tissue samples [34]. These and other features like Aβ variants, oligomeric forms, intra/interchain contacts, peptide fold, arrangement of steric zippers, the parallel/antiparallel orientation of β-strands, amount of β structure, sensitivity to environmental factors or experimental conditions, variable lateral association/supercoiling, and diverse metal binding modes/sites, all contribute to the characteristic polymorphism observed in Aβ fibrils [46–49]. Additionally to ssNMR-derived structural models, recent cryo-electron microscopic models have provided greater insights into the diversity of molecular arrangements that the structure of these aggregates may cover [39–42, 50].

The involvement of Aβ and its oligomeric forms is considered a necessary but insufficient factor responsible for producing the neuropathology in AD, implying that additional cellular and biochemical alterations must exist [51, 52]. Among these, altered metal homeostasis has been implicated as a pathogenic factor in AD [11, 14, 53–56]. Biometals such as copper and zinc promote aggregation of Aβ and are found concentrated within amyloid plaques [57–65]. Moreover, metal ion homeostasis, including zinc, is perturbed in Alzheimer’s disease [66–68]; disturbances on metal ion homeostasis in Alzheimer´s disease is also summarized in refs [56] and [69]. During synaptic transmission, copper and zinc are released into the synaptic cleft, where they are found as Cu^2+^ and Zn^2+^ ions [66, 71]; these metal ions can coordinate to the N-terminal region of Aβ regardless of its state as monomer, oligomer or fibril. It has been proposed that Cu-Aβ species potentially produce reactive oxygen species (ROS), contributing to oxidative stress and neurodegeneration [12, 72]. Even though it is known that the toxicity of oligomeric Aβ is dependent on its metal content [73–77], and that the affinity of biometals for these species may compete with the affinity for other metalloproteins [14, 78, 79], the molecular mechanisms leading to metal-induced aggregation, metal accumulation in amyloid plaques and metal-enhanced cytotoxicity remain unclear.

To gain a better understanding of the role played by metal interactions in the molecular mechanisms underlying the formation of amyloid fibrils, in this work, we addressed the issue by performing molecular dynamics (MD) simulations on a series of atomistic Aβ_42_ amyloid protofibril models in the presence of different ion species. A molecular model is proposed to explain the increased metal ion affinity in amyloid oligomers and fibrils, relating it to the increase of β-sheet structure at the N-terminal domain. The proposed model was tested using disease-modifying variants of the Aβ peptide that contain N-terminal substitutions on key residues. For these variants, significant differences in the number of metal cations accumulated as well as in the amount of N-terminal β-sheets were found, suggesting that the formation of N-terminal β-sheets may be considered as a molecular phenotype modulating the metal-interaction behavior of different Aβ sequences and the concomitant formation of fibrils.

## 2. Computational methods

### Amyloid-β (1-42) protofibril models

**Fig. 2:**
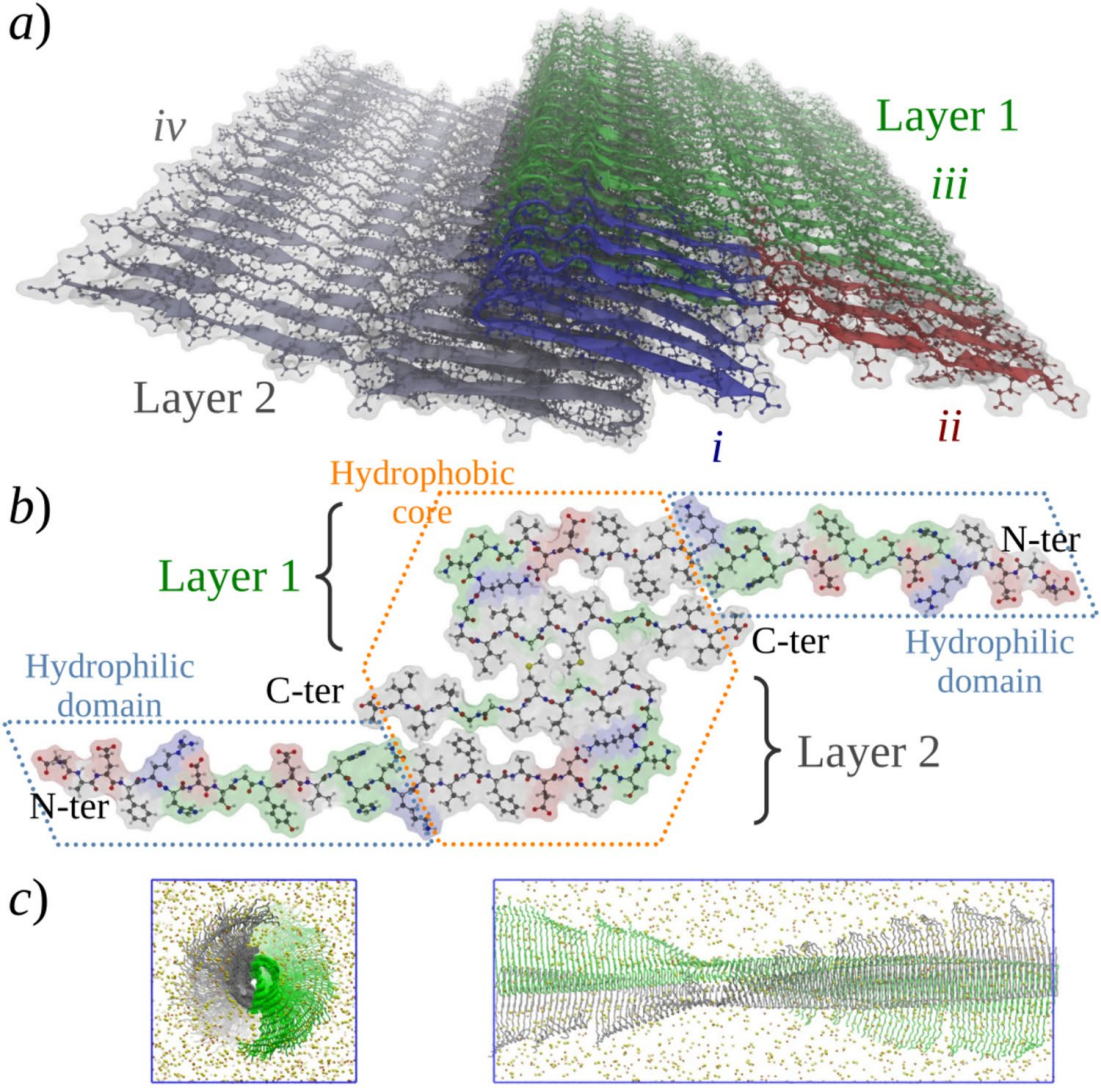
Initial structure of the protofibril models I and V. **a** Building of the 36-strands oligomer (Aβ_42_)_36_ (model I). (*i*) Three chains of Aβ_17−42_ were extracted from the PDB structure 2beg (blue strands). (*ii*) The missing residues 1–16 were added to each chain in an extended conformation (red strands). (*iii*) The three chains were reproduced along the fibril axis to reach 18 monomers (green strands). (*iv*) The whole oligomer was duplicated, rotated over the fibril axis, and assembled to make a *C*_*2*_-pseudosymmetry structure with two layers of Aβ_42_ molecules. **b** Cross-sectional view of two strands from each fibril layer; the amino acid type is highlighted in red for acidic, blue for basic, green for polar, and gray for non-polar residues. **c** Unit cell of the infinite-length (Aβ_42_)_188_ protofibril model V

The structure of the Aβ_42_ protofibril models used in this work are based on the solid state nuclear magnetic resonance (ssNMR) model 2beg [30] from the Protein Data Bank (PDB), considering also specific quaternary-structure interactions observed in ssNMR measurements of Aβ_40_ fibrils [28]. The structure of the N-terminal domain was incorporated into the models as detailed in the Supplementary Information (Section SI-1). The starting model (I), a 36-mer arranged as a 2-layered C_2_-pseudosymmetric protofibril, included an N-terminal (1–16) domain in extended (β) conformation, a non-twisted topology which was spontaneously corrected (twisted) during simulations (see Sect. 3.1), and the same “intra-layer” steric zipper as the original 2beg model along with a new “inter-layer” steric zipper holding the two layers together (see Figs. 2a and b).

As described in Section SI-2 in the Supplementary Information, models II–IV and VI–VII (48-mers) were built from the equilibrated structure of model I; model V (188-mer) was built from model III. An inherited feature in these models was the twisting of the protofibril in the equilibrated model I; considering the equilibrium degree of twisting and the *C*_*2*_ pseudosymmetry of the constructs, model V was built using 94 monomers per protofibril layer that allowed to reach a 180° twist and the periodic reproduction of the structure along the fibril axis. We took advantage of this symmetry and adjusted the water box of model V to allow the periodic boundary conditions to represent an infinite-length amyloid protofibril (Fig. 2c). Table 1 summarizes the composition of the models. The protonation state of all the models corresponds to neutral *p*H with histidine residues protonated at the Nδ of its imidazole ring.

**Table 1:** Composition of the proposed protofibril Aβ_42_ models

Model	Number of Aβ chains	Number of ions			Total number of atoms	Aβ variant
		Na^+^	Cl^−^	Zn^2+^		
I	36	108	–	–	235,881	Human (Wild type)
II	48	–	–	–	302,151	Human (Wild type)
III	48	314	170	–	301,183	Human (Wild type)
IV	48	265	169	48	301,283	Human (Wild type)
V	188	1224	660	–	1,173,246	Human (Wild type)
VI	48	265	169	48	300,107	Human (Tottori)^a^
VII	48	314	170	48	301,117	Rodent (Wild type)^b^

^a^D7N mutation

^b^Amino acid exchanges: R5G, Y10F, and H13R

The water box for models I-VII included different numbers and types of ions to evaluate their effect on the structure of the fibril. Model I included 3 equivalents (eq) of Na^+^ cations to neutralize the protein charge (-3 per Aβ_42_ monomer), while model II was devoid of ions. For models III–VII, the same concentration (0.1 M) of Na^+^ and Cl^−^ ions was considered, i.e., the number of sodium and chloride ions were adjusted to neutralize the final total charge and to reach a salt concentration of 0.1 M, whereas 1 eq of Zn^2+^ (a “soft-sphere” cation per Aβ_42_ monomer with random positions) was added to models IV, VI, and VII. Models VI and VII were intended to assess the stability of N-terminal β-sheets of disease-modifying mutations at the N-terminal region and the effect on the interaction with cations. Model VI contains the familiar AD-promoting Tottori mutation (D7N), whereas model VII corresponds to the non-amyloidogenic Aβ_42_ rodent sequence (R5G, Y10F, and H13R). These considered the same base structure as models II-IV and the same solvent conditions as model IV (1 eq of Zn^2+^ cations and 0.1 M of NaCl). For further details, see Supporting Information section SI-3 and Sect. 3.6.

### 2.2 Molecular dynamics simulations

Equilibration and MD simulations of the models were carried out using the program NAMD2.7[80] and the CHARMM27 force field [81]. Non-bonded interactions were calculated by applying a cutoff of 10 Å and a switching function at 8 Å. Electrostatics were calculated using the particle-mesh Ewald method for periodic boundary conditions [82, 83]. A time step of 2 fs was used to integrate the equations of motion, together with the SHAKE algorithm [84] to keep all bonds to hydrogen atoms rigid. The temperature and the pressure were controlled through Langevin dynamics and the Langevin piston Nosé–Hoover methods as implemented in NAMD [85, 86]. The equilibration protocol consisted of solvent-adaptation, warming up, and density-adaptation stages. For model I, the water molecules were optimized through 3000 conjugate gradient energy-minimization steps and warmed up from 10 to 310 K in a 20 ps simulation, with the protein structure fixed. The system was then released from any structural constraint and minimized for another 3000 steps, followed by a new 100 ps warming simulation and a last NTP run at 310 K and 1 atm for 40 ps. Equilibration of models II-VII followed the same protocol but with longer minimization (5000 steps) and warming up (200–400 ps) runs. The final conditions of the equilibration (310 K, 1 atm) in the NTP ensemble were used in the 100-ns production simulations for all models; in addition, a replica of the simulation of the model I was run for 200 ns to ensure a stationary structure. All structure manipulation, visualization, and trajectory analyses were done using the VMD program. [87] Secondary structure assignments were done using the STRIDE algorithm [88].

## Results and discussions

Several interesting features were observed from the simulations performed on the base model I, most very well recognized by experimental or theoretical evidence. In the following sections, several of these features for all the models are analyzed in detail, and their implications on the molecular mechanisms underlying amyloid aggregation and metal-accumulation are discussed.

### 3.1 Structural features of the amyloid protofibril models

**Fig. 3:**
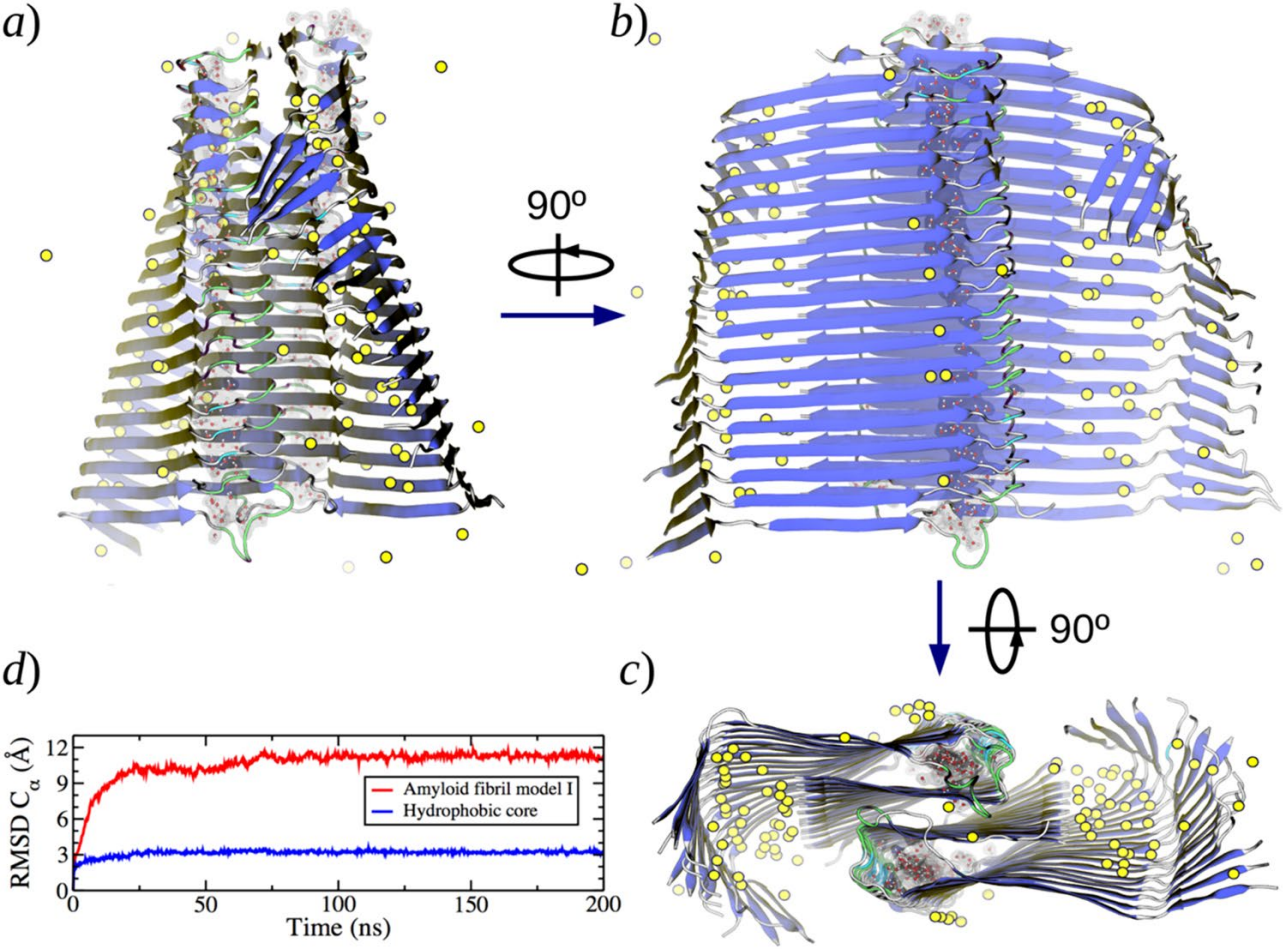
Structure of model I after 100 ns of MD simulation at 310 K and 1 atm. **a**–**c** show a lateral, superior, and frontal views, respectively, of the structure. Yellow circles represent the final positions of Na^+^ ions. The positions of the water molecules inside the hydrophobic core are also highlighted. **d** RMSD Cα vs Time plot considering the whole protofibril model I (red) and only the hydrophobic core (blue) for 200 ns of simulation

After 200 ns of MD simulations, the whole structure of the protofibril model I remained assembled, and most of the features initially adopted were conserved and stabilized. In Fig. 3a–c, the final structures of this model are depicted, highlighting some of the structural transitions and features observed during the simulations, which include twisting of the protofibril structure, formation of water channels within the hydrophobic core, changes in secondary-structure motifs, folding of the N-terminal domain, and distribution of the cations on the surface of the oligomer. These features are described in detail in the following paragraphs.

Although the length of the production simulations may seem too short in comparison to the time needed for amyloid fibrils to form, our models were built from an “already consolidated” fibril structure; thus, the simulation time comprises exclusively the time needed for the structure to adapt and equilibrate to the simulation conditions. Whereas many reported simulations of Aβ have focused on long simulation runs (up to microsecond scale) or enhanced sampling of small oligomers [89–92], in this work, we have focused on larger aggregates to deal with structural features more compatible with consolidated protofibrils rather than small soluble oligomers, with the sacrifice of longer simulation times or exhaustive sampling. In other words, we select larger space scales rather than longer simulation runs. The plot of the root-mean-squared deviation for alpha carbons (RMSD Cα) shown in Fig. 3d confirms that the simulation time was long enough to obtain a stationary structure and to observe important structural transitions (The RMSD Cα plots for the protofibril domains of all models are depicted in Figures SI-1 and SI-2 of the Supplementary Information).

The most evident structural transition in the simulation of model I was the twisting of the aggregate over the protofibril axis (see Figure SI-3a). This feature is common in protein structures with cross-β motifs such as Aβ fibrils [21–23], and has been reported in several MD studies [93–100]. The average angle formed between each pair of monomers plotted against the simulation time, shown in Figures SI-3b, SI-3c, and SI- 3d, quantifies the degree of twisting for all the models. For model I, the twist transition was almost completed within the first 5 ns of simulation, reaching an average angle of ∼2.2°. Considering this degree of twisting, the length required for a longer aggregate to perform a full 180° twist (i.e., crossover distance) is estimated to be ∼39 nm, corresponding to ∼82 Aβ_42_ parallel strands separated by ∼4.8 Å along the protofibril axis (see Table 2).

**Table 2:** Average degree of twisting and morphologic parameters for amyloid protofibril models

Model	Angle per strand (°)	Strands per crossover	Crossover distance (nm)
I	2.2	82	39.4
II	2.1	86	41.6
III	1.8	100	47.4
IV	1.7	106	50.0
V	1.9	95	45.9
VI	1.9	95	45.9
VII	1.9	95	45.9

As it can be seen in Table 2 and in Figures SI-3c and SI-3d, models II–IV, VI, and VII (48-mers) reached a lower degree of twisting relative to model I (a 36-mer); therefore, increasing the size of the oligomer reduces the degree of twisting, as it has been already shown in some MD simulations [93]. Comparing only 48-mers with a different ion environment, model II, containing no ions in the solvent, reached the highest level of twisting (2.1°); models III, IV, VI, and VII showed average angles between strands of ∼1.7°–1.9° per strand, although important fluctuations during the simulations were observed. Thus, the ions present in the solvent also seem to affect the morphology of the protofibril models.

Along the simulations, the models showed degrees of twisting (∼1.5°–2.5° per strand) corresponding to fibril crossover distances in the 35–56 nm range. These results are directly comparable to experimental morphologic characterizations of amyloid fibrils. For instance, a distribution of crossover distances for amyloid Aβ_40_ fibrils grown in phosphate buffer showed a maximum frequency at 50–60 nm [45]; the smallest crossover distances found in that work were ∼30 nm, although even shorter distances (∼25 nm) have been reported [26]. Thus, the degree of twisting obtained in the simulations of the present work is very well aligned with the experimental observations. The capability of reproducing the experimental topologic parameters confirms that the structural features incorporated in the models, all compatible with the definition of the cross-β motif, successfully reproduce the morphology of amyloid protofibrils.

Considering the hydrophobic core region, i.e., sequences 17–42 in both protofibril layers forming steric zippers and stacking four β-sheets (Fig. 1), after production simulations, all the protofibril models conserved the structure of their hydrophobic core assembled and stable, with the two layers of Aβ_42_ peptides paired (intertwined). A systematic study on the size of single/paired-topology oligomers based on the same experimental structure as the present models concluded that a minimum oligomer size of 10–12 monomers would be necessary to obtain stable paired topologies [93]. Our models behaved consequently, in the sense that the number of monomers considered in our models was expected to keep the assigned topology during all the simulation runs, as they did.

**Fig. 4:**
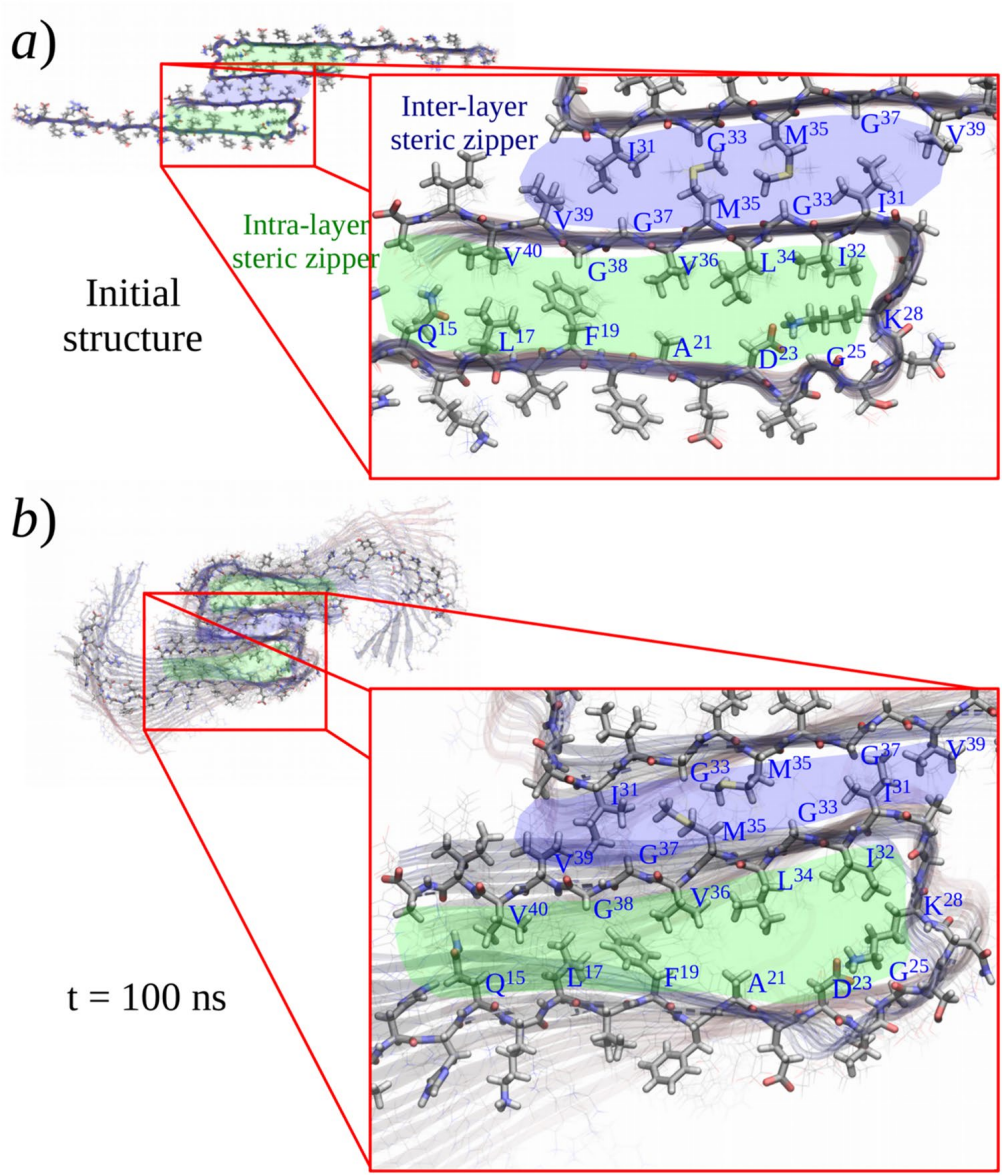
Structure of the steric zippers for a middle Aβ strand in the hydrophobic core of the fibril model I. **a** Initial structure. **b** Twisted structure after 100 ns of MD simulations. In blue, the steric zipper from the interface between the two fibril layers is highlighted, and in green, the steric zipper inside layer

The two types of steric zippers forming the hydrophobic core of the models, i.e., inter-layer and intra-layer steric zippers, in general, conserved the structure of the base models with a few changes. Figure 4 shows the main structural changes observed for model I; a detailed analysis and comparisons can be found in the supporting material (Figures SI-4 and SI-5). For inter-layer steric zippers (blue region in Fig. 4), a compact and dry structure was observed for all the models despite the differences in the protofibril twisting. Although MD simulations have studied different arrangements of the inter-layer steric zipper [93, 94, 98], the general stability observed in all our simulations validates the adopted arrangement. Moreover, this arrangement may explain the reduced aggregation observed for the Met35-sulfoxide Aβ variant [101], as this modification would produce steric hindrance and reduced hydrophobicity within the steric zipper.

Another interesting feature observed in Fig. 4b (green region) is the opening of the intra-layer steric zippers near the turn region, also observed as a broader distribution of distances between alpha carbons in Figure SI-5b. This structural change had two main consequences: (1) distortion of the secondary structure close to the turn region, (see Sect. 3.4), and (2) formation of cavities that allowed the incorporation of water molecules into the hydrophobic core (see also Fig. 3c). This later observation has been reported in MD simulations of Aβ amyloid fibrils [93, 94, 98] or in structures with amphipathic steric zippers [102]. The water molecules in these channels tend to form hydrogen bonds with the peptidic backbone, and even some water molecules were found between the fibril strands. These kinds of interactions have been proposed from 2D infrared experiments. [103]

### 3.2 Structure of the infinite-length (periodic) protofibril model V

**Fig. 5:**
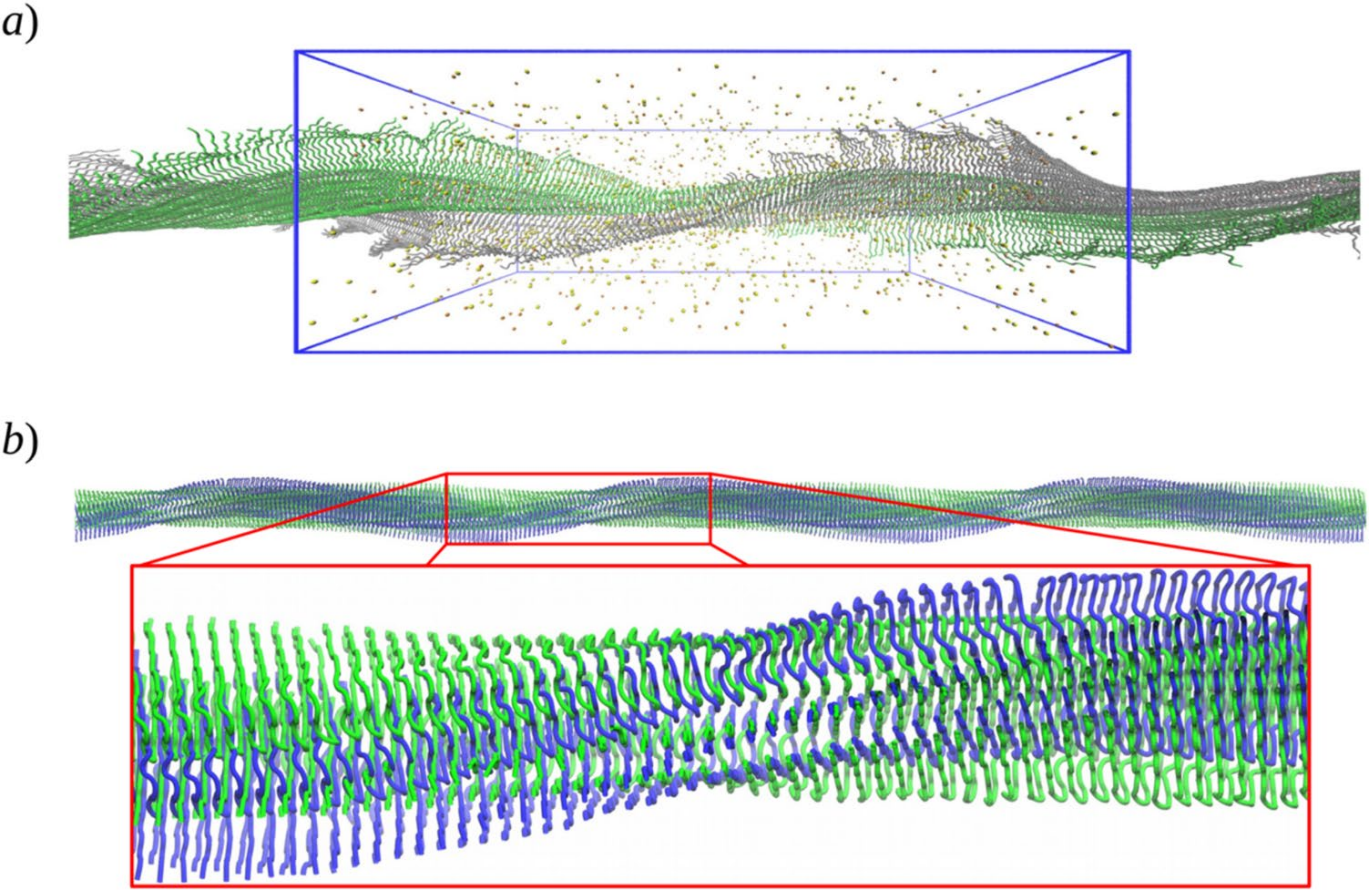
Structure of the periodic amyloid protofibril model V. **a** Perspective view of the final structure (at 100 ns of MD simulation). Yellow and orange spheres represent Na^+^ and Cl^−^ atom positions, respectively. **b** Structure of the hydrophobic core after 100 ns of MD simulation (blue) superimposed to the initial structure (green). The final structure looks arched in the unit cell in comparison to the initial structure

Figure 5a) shows the final structure of model V in the context of its unit cell reproduced periodically. As can be observed, the symmetry of the construct allowed the modeling of an infinite-length protofibril that remained assembled along the simulation.

The most interesting features observed for model V were: (1) the reduction of the N-terminal β-sheet structure, which seems to be an effect of the size of the oligomer (see Sect. 3.3), and (2) the curvature or arching of the protofibril highlighted in Fig. 5b. This arching occurred during the first 30 ns of simulation (Figure SI-1 b in the Supporting information). At this point, the origin of this structural transition is not clear. Two possible factors may be argued: (1) the need for twisting relaxation since, according to our observations, the longer the aggregate, the smaller the degree of twisting, but in model V, it is restricted due to its periodicity. (2) The need for supercoiling: model V is more compatible with a “protofibril” species that tends to associate laterally with one or more similar structural units to form mature supercoiled fibrils, which may be required to form stable linear constructs. Therefore, the arched structure of model V may be more suitable to intertwine with other protofibrils to be stabilized as a straight mature amyloid fibril. It is important to remark that, to our knowledge, this is the first model of an infinite-length amyloid protofibril considering twisted topology; see, for instance, Refs. [94, 95] for analysis of non-twisted periodic models. This model shows interesting differences in relation to finite-length oligomers, and it is worth further analysis in future work as well as for contrasting with experimental observations.

### 3.3 Structure of the N-terminal domain of the amyloid protofibril models

Most of the structural effects observed during the simulations, highlighted in Fig. 3, are associated with the N-terminal region in the protofibril model I. After comparing it with the rest of the models, the structure of this region turned out to be highly affected by the presence of different ions in the solvent. Three features at the N-terminal region of model I stand out: the high amount of β-sheet structure, the folding of the amino-termination, and the regioselective accumulation of cations. These features seem to be related to each other and were found to vary quantitatively when considering different models. As described in deeper detail later, this behavior seems to be related to the high incidence of acidic residues at specific positions of the N-terminal tail, which generates an acidic domain when the aggregate shows β-sheet structure in this region. The high incidence of N-terminal β-sheet opposes several experimental reports, however, the observed elevated stability in most simulations deserves careful analysis. The other two features, namely, the folding of the N-terminal tail and its regioselective accumulation of cations, are processes much less analyzed in the literature due to the difficulty of resolving flexible parts of amyloid fibrils. Thus, the present simulations offer an opportunity to analyze features often invisible to experiments on amyloid aggregates. To better rationalize these features, we are defining a model termed the N-terminal acidic β-sheet domain that allows us to explain the observed features; see below. Due to the relevance of the interaction of metal cations with amyloid fibrils, a detailed discussion of these features and the implication of amyloid aggregation is also done in the forthcoming sections.

### 3.4 Impact of metal ion binding in the structure of Aβ protofibril models

In this and the following sections, those structural transitions and features directly related to the interaction of metal cations with the Aβ_42_ protofibrils in the simulations, including the effect of modifications in the amino acid sequence of the N-terminal domain of the aggregates, are discussed.

**Fig. 6:**
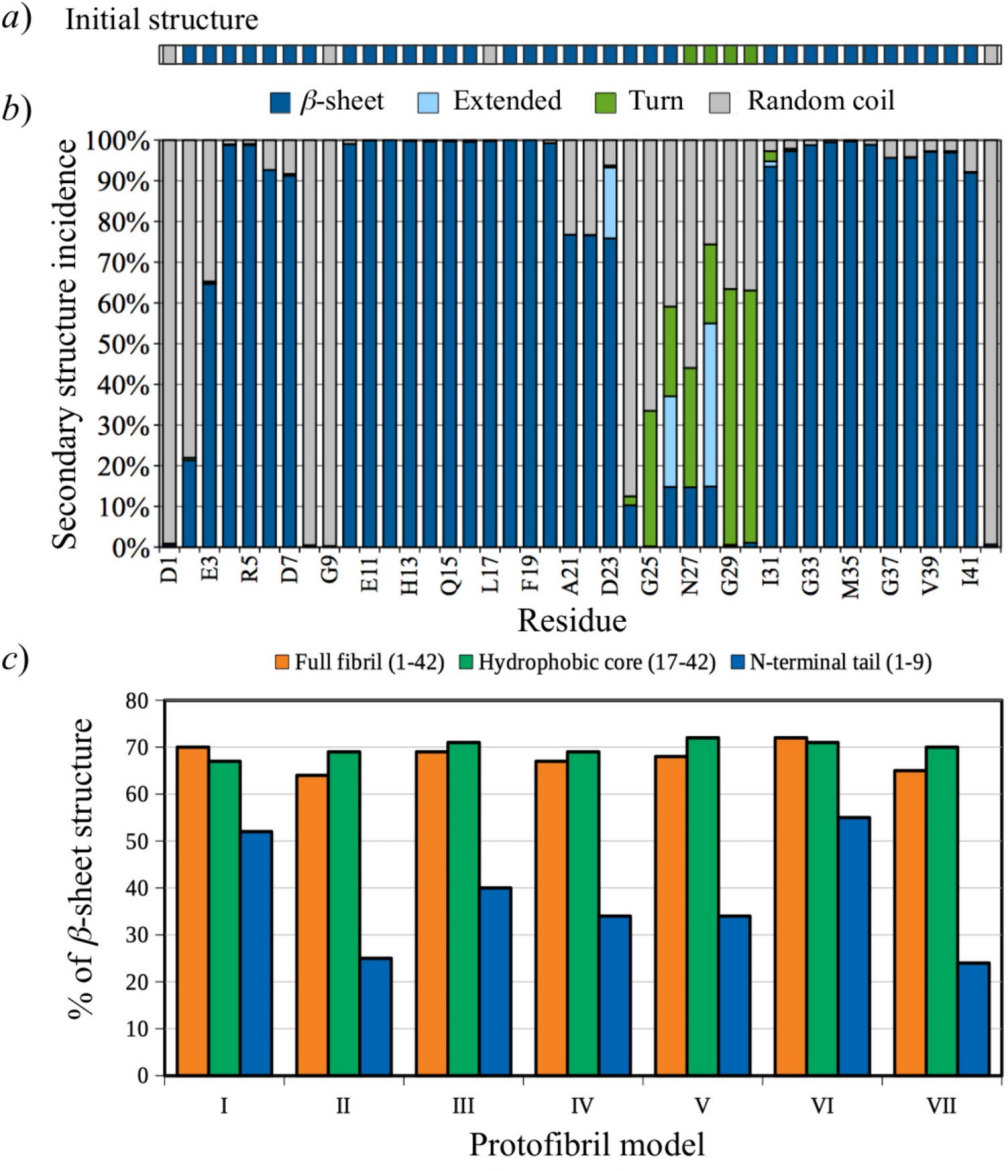
Incidence of secondary structure motifs in Aβ_42_ protofibril models. **a** Initial secondary structure assignment of model I. **b** Average incidence of secondary structure motifs per residue for all strands over the first 100 ns of MD simulation for model I. **c** Incidence of β-sheet structure for different domains of all the protofibril models

From the high amount of β-sheet structure observed in Model I (see Fig. 6a and b) and the comparison with the rest of the models (Fig. 6c and Figure SI-6 in the Supporting Information), it is clear that the secondary structure of the N-terminal region of the aggregates is the most sensitive to the presence of different ions in the models. The amount of β-sheet structure at the hydrophobic core was very stable, which also included part of the N-terminal domain starting at residue 10, showing consistent structure patterns in all the models and good agreement with several reports [23].

At the N-terminal domain, the amount of β-sheet motif was dependent on several factors; considering models that varied their content of ions, model I showed the highest incidence of β structure at the N-terminal domain (52%), whereas the lowest incidence was observed in model II (devoid of ions) (25%). Relative to model I, the increase in the concentration of Na^+^ cations and the addition of Cl^−^ anions in model III decreased the β structure to 40%; further addition of 1 eq of Zn^2+^ cations in model IV decreased even more that motif (34%). Interestingly, with the same ion contents as that in model III, the periodic model V reduced N-terminal β-sheet (34%), indicating that aggregates of smaller size would favor the stability of the N-terminal β-sheet.

Another trend is that the presence of Na^+^ ions seems necessary to favor the N-terminal β-sheet structure. In contrast, the presence of anions or divalent cations would interfere with the formation of this motif. This Na^+^-mediated stabilization of secondary structure is in line with a computational study of the influence of alkali ions on the structure and stability of amyloid-β oligomers, concluding that charge compensation and carboxylate bridging explained the stabilizing effect of alkali ions, which was more evident in smaller oligomers [104]. The observed trend is also consistent with experiments reporting the reduction of β-sheet structure by Cu^2+^ in Aβ_42_ fibrils [105] or by Cu^2+^ or Zn^2+^ in both Aβ_40_ and Aβ_42_ [106]. Metal ions affect the final morphology of amyloid fibrils, forming less defined or even non-fibrillar aggregates [63, 77, 106]. Moreover, copper-chelating agents yield less branched and elongated fibrils, as compared to those grown in the presence of the metal [107], which indicates that copper may induce a different aggregation pathway for Aβ.

Although, the structure of the N-terminal region in model I was initially built in extended (β) conformation, this motif was expected to be unstable in a relatively long production simulation, as this region was generally reported as a random coil [30]. However, even after 200 ns of MD simulation, the amount of N-terminal β-sheet structure did not show any decreasing trend during the simulation of model I (see Figure SI-7 in the Supporting Information). On the other hand, several experiments provide evidence of forming N-terminal β-sheets in Aβ aggregates [39, 106, 108–110]. Moreover, several structural models have been solved with well-defined N-terminal tails with stable β-sheet motifs [34, 36, 38, 39]. Interestingly, it has been suggested that N-terminal β-sheets may play a role in the amyloid aggregation pathway, favoring protofibril species over mature fibrils [110]. The consolidation/distortion of β-sheets in the N-terminal domain of amyloid oligomers may thus be characteristic of different metastable forms of oligomeric Aβ species resulting from different aggregation pathways.

**Fig. 7:**
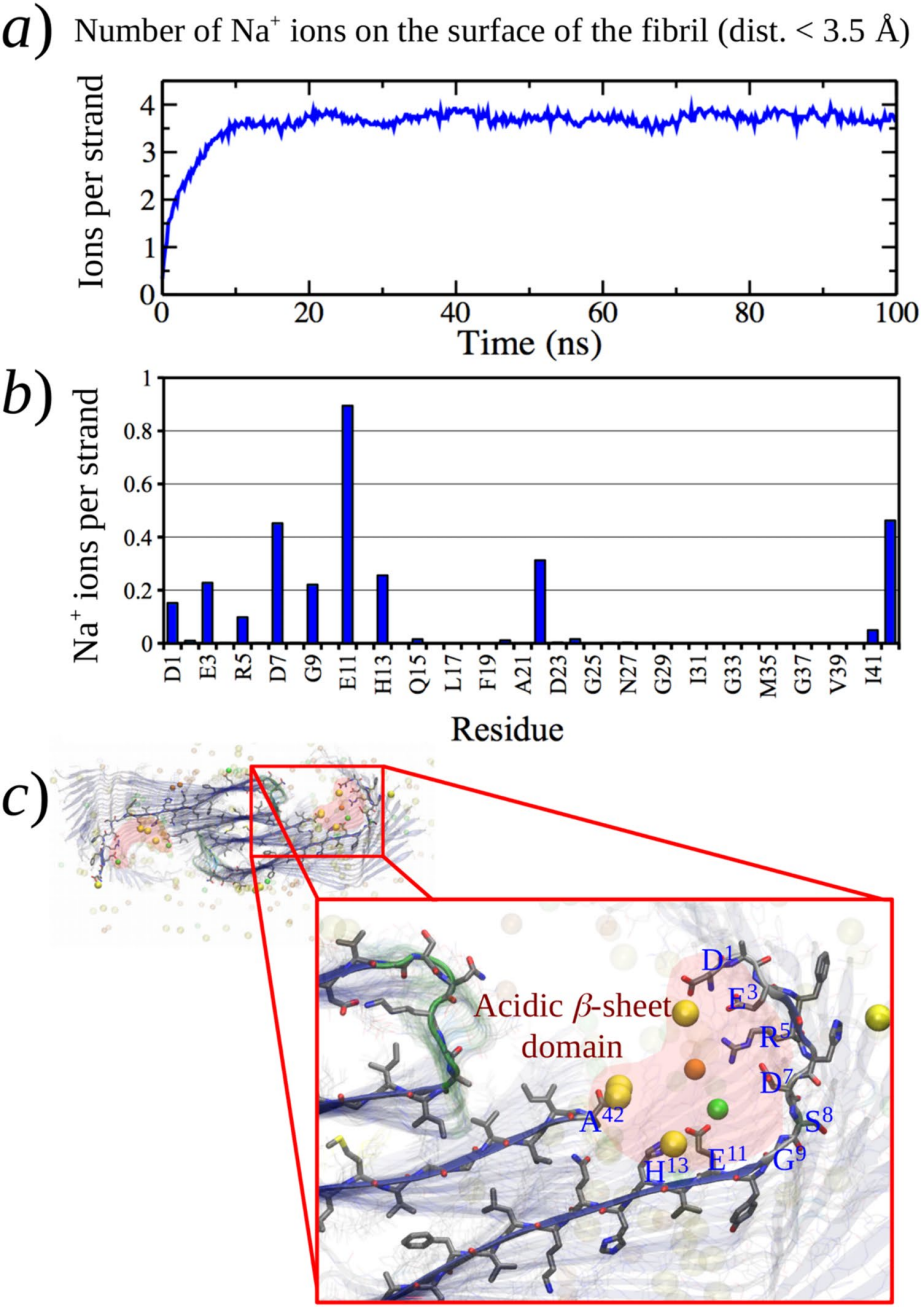
Ions on the surface of protofibril models within 3.5 Å from the protein. **a** Number of sodium ions against time for model I. **b** Average number of sodium ions per strand and per residue for model I. **c** Structure of the N-terminal domain of one strand of model IV. Na^+^ (yellow), Cl^−^ (orange), and Zn^2+^ (green) ions are shown

The interaction of the ions with the protofibril models was analyzed to understand the origin of the regioselective accumulation of cations in the structural features observed at the N-terminal domain of the models. As depicted in Fig. 7a), the Na^+^ ions saturated the surface of the protofibril model I during the first ∼10 ns of simulation. We quantified the average number of ions in contact with each Aβ_42_ residue (within 3.5 Å), finding that the residues interacting with Na^+^ ions (in addition to Glu22 and Ala42) were found at the N-terminal domain and exclusively at odd positions (Fig. 7b). This regioselective interaction may be explained in terms of the β-sheet structure preserved at the N-terminal domain of model I, which keeps the side chains of the acidic residues (Asp1, Glu3, Asp7, and Glu11) on a single face of the N-terminal β-sheet. This produces a region predominantly rich in acidic residues, capable of attracting and concentrating cations from the media, and that can be described as an N-terminal *acidic β-sheet domain* (Fig. 7c).

Four conditions drive the formation of the *acidic β-sheet domain*: (1) The conserved β-sheet structure at the N-terminal domain that properly orients acidic side chains; (2) the structure and fold of the hydrophobic core making the peptidic carboxylate of Ala42 reachable to the N-terminal domain; (3) the folding of the N-terminal domain toward the hydrophobic core allowed by a bend-like region at residues Ser8 and Gly9; (4) the Aβ primary sequence by itself, specially at the N-terminal tail. Indeed, the arrangement of the N-terminal acidic residues along with Arg5 and His13, as shown in Fig. 7c, strongly suggests a functional role of the N-terminal sequence of the Aβ peptide. Combining these features to form this acidic region establishes a connection between the accumulation of cations and the formation of the N-terminal β-sheet, a process dependent on the aggregation of the Aβ protein. Additionally, some experimental reports support the formation of the *acidic β-sheet domain*. For instance, ssNMR measurements have shown the participation of carboxylate groups from glutamate residues and the C-terminus of Aβ_40_ fibrils in Cu.^2+^ coordination [111]. Moreover, a kink or bend-like structure, consistent with the observed bending at Ser8 and Gly9, has been reported. [18, 108]

**Fig. 8:**
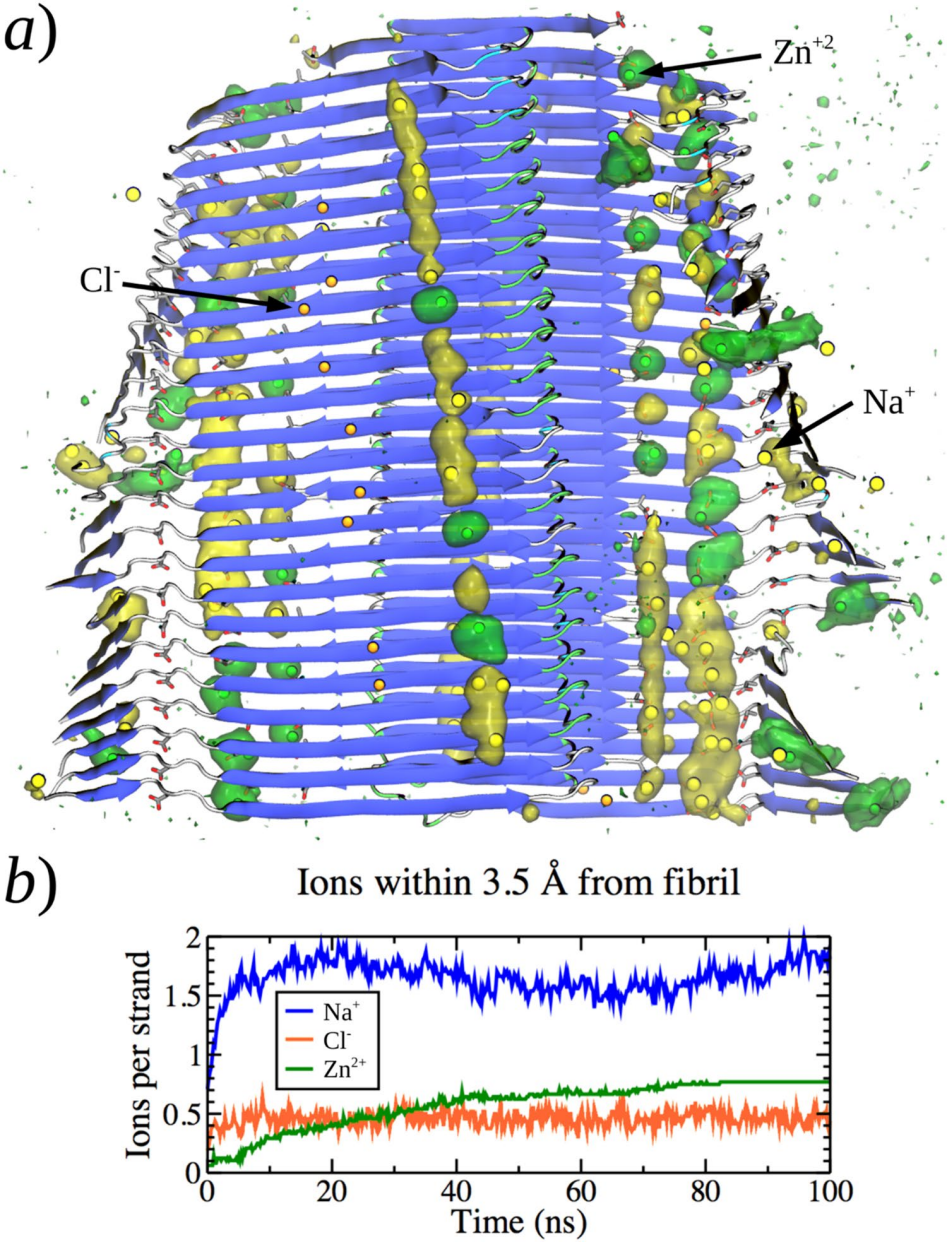
Interaction of ions with fibril model IV. **a** Ion density isosurface (isovalue = 0.07, considering the last 20 ns of simulation) for Zn^2+^ (green) and Na^+^ (yellow) ions over the final fibril structure (blue β-strands). **b** Average number of ions per fibril strand at less than 3.5 Å. Yellow, orange and green spheres represent Na^+^, Cl^−^ and Zn^2+^ ions, respectively

In the case of model IV incorporating Zn^2+^ cations, despite the reduction of the N-terminal β-sheet, the regioselective accumulation of cations was also observed (Fig. 8). Actually, the ion-density isosurfaces in Fig. 8a) show that the specific interaction of Zn^2+^ affected the secondary structure and fold of the N-terminal domain. Figure 8b shows that both Na^+^ and Zn^2+^ cations seem to compete for the *acidic β-sheet domain* of the model. Although Na^+^ ions saturated the protofibril surface within 20 ns, the number of interacting Zn^2+^ ions slowly raised during 80 ns of this simulation, displacing the necessary amount of sodium.

The formation of the *acidic β-sheet domain* implies folding or bending of the N-terminal domain toward the hydrophobic core. We quantified this folding for each model as the distribution of distances between N- and C-terminal alpha carbons through the radial pair distribution function g(r) shown in Figure SI-8. For the models varying the content of ions (I–IV), a maximum close to 30 Å was observed except for model II, where the maximum was shifted up to 43 Å. This suggests that the absence of ions in model II produced more extended (less folded) strands at the N-terminal domain, probably due to the repulsion of negative charges from the acidic residues at the *acidic β-sheet domain*. The difference in the folding of the models containing cations relative to model II shows the effect of the cations in shielding the negative charge at the *acidic β-sheet domain*.

### 3.5 Implications on the N-terminal *acidic β-sheet domain* in metal binding

The described molecular mechanisms at the N-terminal domain of the protofibril models have important implications in the coordination chemistry of amyloid fibrils, since the residues forming the *acidic β-sheet domain* may also participate in metal coordination. The involvement of Aβ N-terminal residues such as Asp1, His6, Glu11, His13, and His14 in metal coordination either as monomers or oligomers has been described [111–119]. Considering that histidine residues are known to be effective anchoring ligands, specially in multi-histidine peptides forming macro-chelate rings [120], metal coordination involving histidine together with aspartate and/or glutamate residues will impose structural restrictions that would enhance the observed reduction of the N-terminal β-sheet, without necessarily affecting the hydrophobic core of the fibril. Copper coordination to N-terminal residues of the protofibril was already studied in models of the peptide Aβ1-17 in complexes with Cu^2+^ through all-electron density functional theory (DFT) calculations from one of the strands of model I [121]. Although several binding modes were found upon copper coordination, the extended conformation from residues 11 to 17 was almost unaffected (see Figure SI-9 in the Supplementary Information). This supports the fact that the effects of the metal ions on the amyloid fibril structure originated at the N-terminal domain, which is also compatible with the recently proposed N-terminal hypothesis for AD [122].

### 3.6 Molecular phenotype of Aβ_42_ variants: effect of N-terminal mutations on metal binding and β-sheet stability

Together with the turn region, the N-terminal domain has been shown to contain the major structural differences in Aβ oligomers relative to mature fibrils; moreover, both regions contain most of the Aβ mutations relevant in AD [123]. Thus, these regions are of particular interest. Considering also the formation of the *acidic β-sheet domain* and its tendency to accumulate cations, we aimed to investigate the effect of mutations in the N-terminal domain on the metal-accumulation properties of this region considering known disease-modifying variants of Aβ. Particularly, mutations in residues forming the *acidic β-sheet domain* are expected to have an important effect in the binding of ions and the stability of the N-terminal β-sheet structure according to the molecular mechanisms observed on the protofibril models described in previous sections.

As already argued, the participation of acidic residues (Asp1, Glu3, Asp7, and Glu11) and the bending of Ser8 and Gly9 are key to forming the *acidic β-sheet domain*. Residues Arg5 and His13 have interesting implications too. Arg5 was observed to assist in folding the N-terminal domain and consolidating the N-terminal β-sheet, whereas His13 would have a role in metal coordination. These residues, along with others forming the *acidic β-sheet domain*, are known to be relevant in disease-modifying natural variants of the Aβ sequence. For instance, the rodent-Aβ sequence (H13R, R5G, and Y10F substitutions), which rarely forms amyloid plaques (i.e., a protective variant) [124], was shown to form amyloid plaques with reduced metal binding abilities relative to human Aβ [125]. These observations may be related to the highlighted roles of Arg5 and His13. Moreover, His13 is a crucial residue in the Zn^2+^-induced aggregation of Aβ [126]. In the context of the present analysis, the regioselective accumulation of cations may be proposed to be a preamble favoring Zn^2+^ coordination to His13 in the *acidic β-sheet domain*, which affects the aggregation pathway and thus would be altered in the rodent sequence, explaining the behavior of this variant. Interestingly, isothermal titration calorimetry (ITC) measurements on rat Aβ_1-16_ peptide in the presence of Zn^2+^ may provide insights into such role for His13; [127] in those experiments only residues His6 and His14 of the rat sequence were shown to be involved in Zn^2+^ coordination, forming peptide dimers complexed with zinc in an arrangement that could hinder peptide aggregation [127]. In contrast, metal binding to His13 in human Aβ would promote coordination to Asp and Glu residues within the *acidic β-sheet domain*. Other substitutions found in the *acidic β-sheet domain,* such as D7N, known as the Tottori familial AD mutation [128], are known to modify the aggregation properties of this (AD-promoting) Aβ variant [129, 130]. From the analysis performed on models I-V, specially regarding the regioselective accumulation of cations at the acidic β-sheet domain and the elevated stability of the N-terminal β-sheet, we expected that mutation of residue Asp7 to asparagine in the Tottori variant would modify the metal-accumulation behavior in the studied models. We also chose the rodent-Aβ sequence as a second variant associated with an opposite phenotype relative to the Tottori to gain further insights into the roles of N-terminal residues.

To evaluate such roles, we built models VI and VII based on the Tottori and rodent sequences of the Aβ_42_ protein, respectively, which were subjected to the same simulation conditions and ion contents as model IV, acting as reference (human Aβ sequence). Table 3 shows the time-averaged incidence of N-terminal tail β-sheet and the number of cations in the surface of the protofibril models, whereas Figures SI-10 and SI-11, show the detailed time evolution of each property.

**Table 3:** Average of properties measured for the N-terminal domain of protofibril models of Aβ_42_ variants

Property	Human Aβ (model IV)	Tottori variant (model VI)	Rodent Aβ (model VII)
N-ter tail (1-9) β-sheet (%)	34	55	24
Maximum of Nt-Ct g(r) (Å)^a^	32	8, 27^b^	6
Zn^2+^ ions per strand	0.49	0.34	0.30
Na^+^ ions per strand	1.20	0.72	1.49
Zn^2+^/Na^+^ ratio	0.41	0.47	0.20
Cations per strand	1.69	1.06	1.79
Cl^−^ ions per strand	0.45	0.46	0.41
Total ion charge per strand	1.73	0.94	1.68
Total protein charge per strand	− 3	− 2	− 3
Total charge for residues 1, 3, 5, 7, 11, 13, and 42^c^	− 4	− 3	− 4

^a^Refers to the position (distance) where the maximum of the distribution of N- to C-terminal distances is located. A small value corresponds to collapsed or folded N-terminal strands, while large values correspond to extended strands

^b^Two maxima were observed

^c^Residues 1, 3, 5, 7, 11, 13, and 42 are located within the acidic β-sheet domain

From Table 3, it can be observed that the substitutions at the N-terminus of the Aβ sequence produced important differences in the amount of N-terminal β-sheet structure, the folding of the N-terminal tail, and the number of cations accumulated at the *acidic β-sheet domain*. The AD-promoting D7N variant (model VI) maximized the N-terminal β-sheet amount, whereas the aggregation-preventive rodent sequence (VII) minimized it. The amount of N-terminal β-sheet may thus be considered a molecular phenotype for the aggregates of these variants. These observations are consistent with experimental measurements of secondary structure for the Tottori variant that, additionally, were associated with the increased toxicity of these aggregates [130]. Moreover, replica-exchange molecular dynamics simulations on D7H, D7N, and H6R mutants of the Aβ peptide showed an increase in the amount of β structure and enhanced the turn at Ser8 and Gly9. [131] The distortion of the N-terminal β-sheet in the rodent sequence may be directly associated with its reduced aggregation, whereas the reduced metal load may be associated with the H13R substitution located in the *acidic β-sheet domain*. The N-terminal β-sheet distortion in model VII resulted in collapsed strands, as reflected by the distributions of N-ter to C-ter distances (see Figure SI-8).

The average amount of each ion in contact with the *acidic β-sheet domain* (within 3.5 Å) shows that among the three models in Table 3, both the amount of Zn^2+^ and Na^+^ cations varied while the amount of Cl^−^ remained almost the same. Zn^2+^ cations were more crowded in model IV; for model VI, the amounts of Zn^2+^ and Na^+^ decreased relative to IV, whereas in model VII, Zn^2+^ decreased and Na^+^ increased. The Zn^2+^/Na^+^ ratio reveals that the proportion of these cations is almost the same for models IV and VI and that model VII became selective for Na^+^ cations. Therefore, although reducing the negative charge in the D7N variant reduces the number of both cations, the selectivity for each cation depends on the Aβ sequence and probably on the amount of the N-terminal β-sheet motif.

The results regarding the amount of N-terminal β-sheet are consistent (in terms of the associated phenotypes) with the effects on the N-terminal domain of the A2T and A2V mutations studied in Aβ_42_ dimers [132], the AD-protective A2T variant diminishes N-terminal contacts, whereas the AD-causative A2V variant and the wild-type sequence promotes them. From the present study, the AD-protective rodent sequence reduces the N-terminal β-sheet (i.e., diminishes N-terminal contacts); the human Aβ sequence and the AD-causative D7N variant increase the N-terminal β-sheet. In our models, the AD-preventive variant also loads less Zn^2+^ than the AD-causative. The D7N and other charge-modifying variants of the Aβ sequence may have a direct effect on the solubility of the aggregates, which would be added to the putative charge-reducing effect upon zinc coordination to acidic Aβ residues [11]. An increased hydrophobicity may have an effect on the aggregation behavior of amyloid aggregates as well as in the interaction with biological membranes. These effects may have consequences in the neurotoxicity of amyloid oligomers and metal-mediated neurotoxicity.

## 4. Conclusions

The time evolution of the structure and other properties of the different Aβ_42_ protofibril models in response to different simulation conditions described here in the presence of metal ions may be interpreted in the context of the mechanisms of metal accumulation in amyloid plaques and on metal-induced aggregation and cytotoxicity. The regioselective accumulation of cations through the formation of the proposed *N-terminal acidic β-sheet domain* provides by itself a molecular mechanism that directly resembles the observed accumulation and co-localization of metals within amyloid plaques in brain tissue samples, together with the increased metal affinity for oligomeric forms. The described fold of the N-terminal domain produces confinement of the N-terminal hydrophilic residues together with the attracted cations, which has the potential of increasing the hydrophobicity of the aggregate, an effect further enhanced by the reduction of the Aβ charge that metal coordination to acidic residues would produce. The increase in hydrophobicity has implications in Aβ aggregation and in the interaction with membranes (i.e., affecting their permeability); both consequences may be related to the neurotoxic properties of amyloid aggregates, which are in agreement with the enhanced amyloid aggregation and toxicity promoted by metals. It was also suggested that the distortion of the N-terminal β-sheet structure by metal ions might be related to the change in the pathway of Aβ aggregation. Since the consolidation of β-sheet structure requires oligomerization in Aβ, and the N-terminal β-sheet tends to attract cations, amyloid aggregation may respond to metal dyshomeostasis. These effects may be connected to the high β-sheet content characteristic in amyloid fibrils. The molecular mechanisms here described and contrasted with experimental reports enhance our understanding of the molecular-level processes relevant to the aggregation of Aβ peptides that form AD amyloid plaques, and aid in the comprehension of the role that metal ions such as zinc may play in such processes, particularly, the role that the Aβ N-terminal sequence and its interaction with metals plays.

### Supplementary Information

Below is the link to the electronic supplementary material.

Supplementary file1 (DOCX 9659 KB)

## Data Availability

The data encompassing input/output files, scripts, raw analysis data, and visualizations generated throughout this study are openly accessible upon direct inquiry to the authors. We welcome requests for any supplementary materials utilized in our computational investigations.
