# Supplementary material for: An N-terminal acidic β-sheet domain is responsible for the metal-accumulation properties of amyloid-β protofibrils: a molecular dynamics study
Source: J Biol Inorg Chem. 2024 May 29;29(4):407–25. doi: 10.1007/s00775-024-02061-1 (PMC11186886; doi:10.1007/s00775-024-02061-1)
Supplement: Supplementary file 1 — Supplementary file1 (DOCX 9659 KB) [file 775_2024_2061_MOESM1_ESM.docx]

**Supplementary Information**

An N-terminal acidic β-sheet domain is responsible for the metal-accumulation properties of amyloid-β protofibrils: A molecular dynamics study

Carlos Z. Gómez-Castro^1^*, Liliana Quintanar^2^* and Alberto Vela^2^*

^1^Conahcyt - Universidad Autónoma del Estado de Hidalgo, km 4.5 Carr. Pachuca-Tulancingo, Mineral de la Reforma, Hidalgo, 42184, Mexico.

^2^Department of Chemistry, Cinvestav, Av. Instituto Politécnico Nacional 2508, San Pedro Zacatenco, Gustavo A. Madero, 07360, CDMX, Mexico

^*^Corresponding author(s). E-mail(s): czgomez@conahcyt.mx;

lilianaq@cinvestav.mx; avela@cinvestav.mx;

**Contents:**

**Section SI-1. Building of the amyloid beta (1-42) protofibril model I.**

**Section SI-2. Building of the amyloid beta (1-42) protofibril models II-V.**

**Section SI-3. Building of the amyloid beta (1-42) protofibril models VI and VII.**

**Figure SI-1. Mass-weighted root-mean-square-deviations for alpha-carbon atoms of the structural domains of the amyloid protofibril models I-V. a) Full sequence (residues 1-42, all fibril strands). b) Hydrophobic core (residues 17-42). c) N-terminal domain (residues 1-16).**

**Figure SI-2. Mass-weighted root-mean-square-deviations for alpha-carbon atoms of the structural domains of the amyloid protofibril models III, VI, and VII. a) Full sequence (residues 1-42, all protofibril strands). b) Hydrophobic core (residues 17-42). c) N-terminal domain (residues 1-16).**

**Figure SI-3. Twisting of the amyloid protofibril models. a) Initial and final side views of the structure of model I. b) Plot for the average angle between strands against the simulation time per protofibril layers of model I. The inset of b) shows the plot of the global angle for the whole model against time, i.e. the relative angle between the first and the last strands of the fibril. c) Average angle between strands for models I-V. d) Average angle between fibril strands of models IV, VI, and VII.**

**Figure SI-4. a), b) and c) show the frontal, longitudinal and transversal views of fibril model I highlighting the relative positions of the alpha-carbon atoms of the residues forming the steric zippers in the hydrophobic core. d) Distribution of distances between alpha-carbon atoms on the interlayer steric zipper in model I. e) Distribution of distances between alpha-carbon atoms in the intralayer steric zippers in model I.**

**Figure SI-5. Distribution of distances in the inter- (a) and intra-layer (b) steric zippers for the amyloid fibril models I-V, considering 100 ns of MD simulation.**

**Figure SI-6. Incidence of protein secondary structure motifs per residue for the amyloid fibril models I-VII.**

**Figure SI-7. Incidence of N-terminal (1-9) beta-sheet structure against time for protofibril models I-V. Model I’ represent an independent simulation (replica) of model I that was run for 200 ns.**

**Figure SI-8. Radial pair distribution function between Cα atoms from N-terminus (blue spheres) and C-terminus (red spheres) for the protofibril models during the last 5 ns of simulation. a) Models I-V. b) Models IV, VI, and VII.**

**Figure SI-9. Structure of Cu^2+^-Aβ17 complexes (green strands) superimposed to the structure of the amyloid fibril model I (blue strands).**

**Figure SI-10. Incidence of N-terminal (1-9) beta-sheet structure against time for protofibril models IV, VI, and VII.**

**Figure SI-11. Number of Na^+^ (a) and Zn^2+^ (b) cations in contact (within 3.5 Angstroms) with the N-terminal acidic β-sheet domain of amyloid protofibril models IV, VI, and VII.**

**References**

**Section SI-1. Building of the amyloid beta (1-42) protofibril model I.**

Out of five chains (A-E) contained in the PDB structure 2beg, three middle chains (B-D) were extracted. These correspond to a trimer (Aβ_17-42_)_3_ aligned in a structure consistent with the cross-beta motif (see ref 1) and would be the base of the hydrophobic core of our models. Note that the second model (out of the 20 best NMR models in structure 2beg) was chosen because this case contained a consistent β structure for the strands B-D. The N-terminal portion (residues 1-16) was built and added to each strand of the extracted trimer in the same extended conformation as the contiguous residues. To achieve this, we extracted the backbone coordinates (including beta-carbons if present) of the portion 17-24 of each chain of the trimer, translated the coordinates to the N-terminal side of residue 17, deleted the appropriate atoms to form a peptidic bond between residues 17 and 24, and performed the necessary mutations (and renumbering) according to the Aβ_9-16_ sequence with the aid of the psfgen program included in VMD.^2^ The portion 1-8 was appended using an analogous procedure. It is worth noting that the structure of the 17-24 portion used to build the N-terminal domain was in extended conformation forming a β-sheet in the original model 2beg, thus the added N-terminal portions conserve the structural features and extends the N-terminal β-sheet. The resulting (Aβ_1-42_)_3_ trimer was then reproduced and translated repeatedly along the fibril axis to generate an 18-mer. This (A*β*_42_)_18_ oligomer (layer 1) was duplicated to provide a second layer of A*β* molecules that was rotated over the fibril axis and docked to layer 1 forming a *C_2_*-pseudosymmetry aggregate (A*β*_42_)_36_. This docking followed specific quaternary-structure interactions observed from ssNMR measurements for A*β*_40_ fibrils, i.e., the direct interaction of residues Gly33 and Met35 from each fibril layer.^3^ This model was optimized for 500 steps to correct steric clashes and immersed in a box with 71,175 TIP3 water molecules and 108 randomly placed Na^+^ ions to neutralize the total charge. The model was submitted to an equilibration protocol and a long MD production simulation; the equilibrated structure was used to build models II-V (see next Section).

**Section SI-2. Building of the amyloid beta (1-42) protofibril models II-V.**

Models II-IV were built from the equilibrated structure of model I after 50 ns of an MD simulation at 310 K and 1 atm. These models consisted of a 2-layered *C_2_* 48-mer (24 x 2 strands) that inherited most of the structural features adopted by model I, particularly the degree of twisting. The construct was built using the middle 12-mer, i.e. the innermost 6x2 strands of the total 18x2 strands extracted from model I, reproduced, and repeatedly aligned to form an (A*β*_42_)_48_ aggregate. This was done considering an extra pad of two “dummy” strands on each side of the 12-mer (along the protofibril axis) to perform structural alignment of the oligomer repeats (minimizing the RMSD of alpha-carbons from residues 17-41 in dummy strands), and then deleting the dummy strands. Figure SI-2A *a*) illustrates the alignment of model I to build a bigger model, where red strands are used for alignment. The resulting (A*β*_42_)_48_ oligomer was used in models II-IV (same protein structure) and differ only in the kind of ions added to the solvation box, i.e., model II contained no ions, and therefore the protein charge was not neutralized, model III contain a concentration of Na^+^ and Cl^-^ ions of 0.1 M neutralizing the total charge of the system, whereas model IV contained the same amount of NaCl plus 1 eq of Zn^2+^ cations (48 ions), neutral charge too.


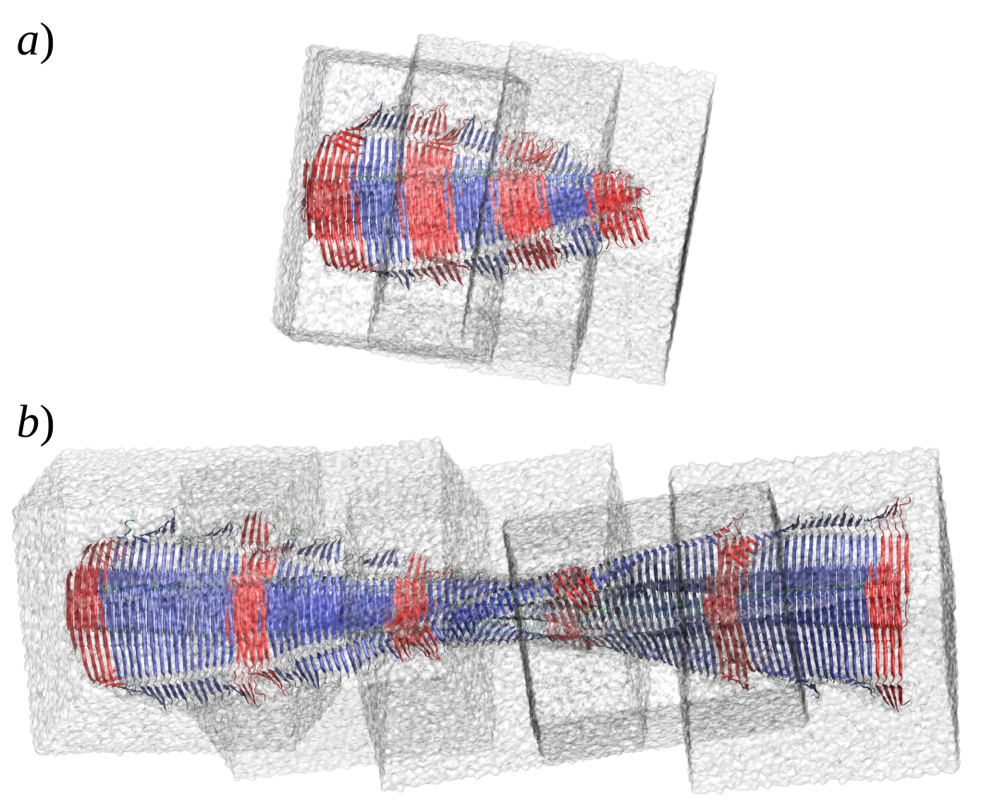


Figure SI-2A. Example of the alignment of pre-equilibrated oligomers to build a bigger aggregate. *a*) Show three repetitions of model I, aligned through the red strands (by alpha carbons of residues 17-42). *b*) Five repetitions of model III aligned through the red strands to build model V.

For model V, a procedure similar to that used to generate models II-IV was followed with the difference that the equilibrated structure of model III was used as base structure after ~32 ns of MD simulation at 310 K and 1 atm. Then, 40 middle strands of model III were repeated and aligned to form the 2-layered *C_2_* (A*β*_42_)_188_ oligomer that inherited the degree of twisting of its base model and thus allowed it to reach a global twist angle of ~180° (see Figure SI-2A *b*). This was considered to model a periodic construct representing a twisted infinite-length protofibril model (see Section 3.9 of the main text). Model V considered the same NaCl concentration as model III.

**Section SI-3. Building of the amyloid beta (1-42) protofibril models VI and VII.**

The building of models VI and VII followed the same procedure as models II-IV, with the difference that in the protein assembly step (with the program psfgen), the substitution Asp7 to Asn in model VI, and Arg5 to Gly, Tyr10 to Phe, and His13 to Arg for model VII, were applied through the program psfgen. The contents of ions, the equilibration protocol, and the production simulation were the same as in model IV.


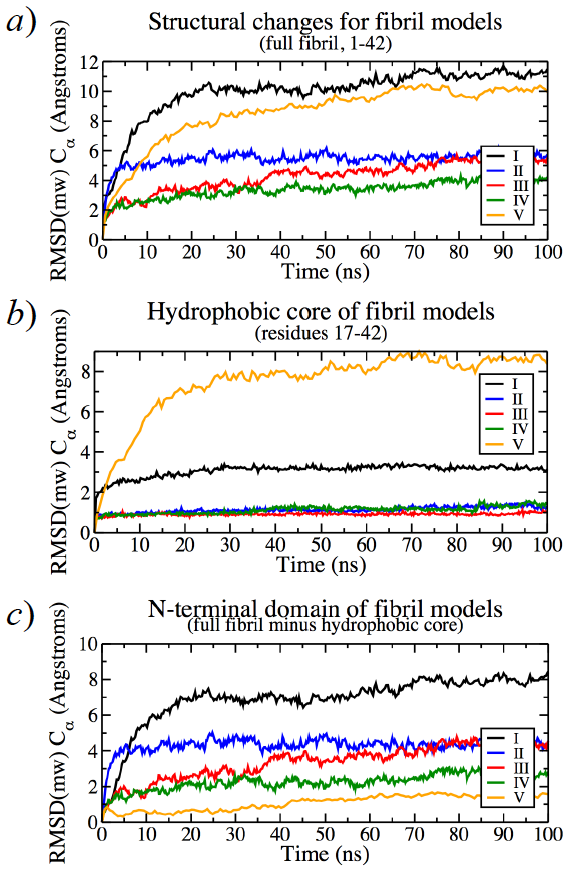


**Figure SI-1.** Mass-weighted root-mean-square-deviations for alpha-carbon atoms of the structural domains of the amyloid protofibril models I-V. *a*) Full sequence (residues 1-42, all fibril strands). *b*) Hydrophobic core (residues 17-42). *c*) N-terminal domain (residues 1-16), obtained by subtracting the data in *b*) from that in *a*). The major structural transitions found correspond to the twisting of the flat initial structure of model I, the folding of the N-terminal domain for the same model, and the arching of the protofibril model V. Note: In general, the same atoms used for fitting (alignment) were used in RMSD calculation, only in few cases alpha carbons for residues in the hydrophobic core (17-42) were used for fitting while the RMSD was calculated for alpha carbons of residues at the N-terminal domain. In general, only alpha carbons of each residue in each protofibril strand were considered in the calculation, sampling every 50 ps for time averages.


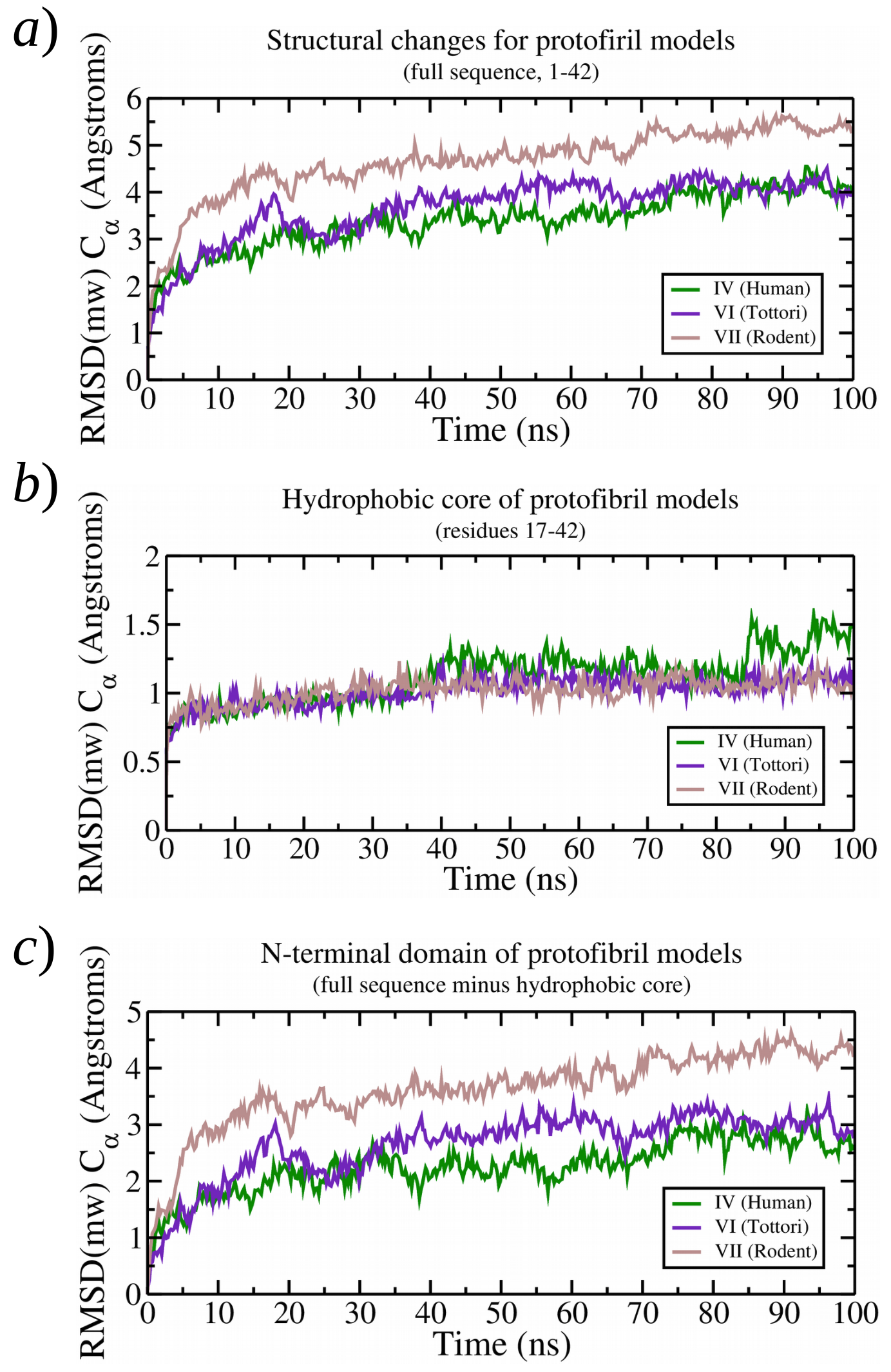


**Figure SI-2.** Mass-weighted root-mean-square-deviations for alpha-carbon atoms of the structural domains of the amyloid protofibril models III, VI, and VII. *a*) Full sequence (residues 1-42, all protofibril strands). *b*) Hydrophobic core (residues 17-42). *c*) N-terminal domain (residues 1-16), obtained by subtracting the data in *b*) from that in *a*). The major structural transition was observed for the N-terminal domain of model VII that corresponds to the disease-protecting rodent variant of the A*β*_42_ sequence. The disease-promoting Tottori variant showed similar transitions as the wild-type variant at the N-terminal domain. The three variants showed very similar behavior at the hydrophobic-core domain of the protofibril models.


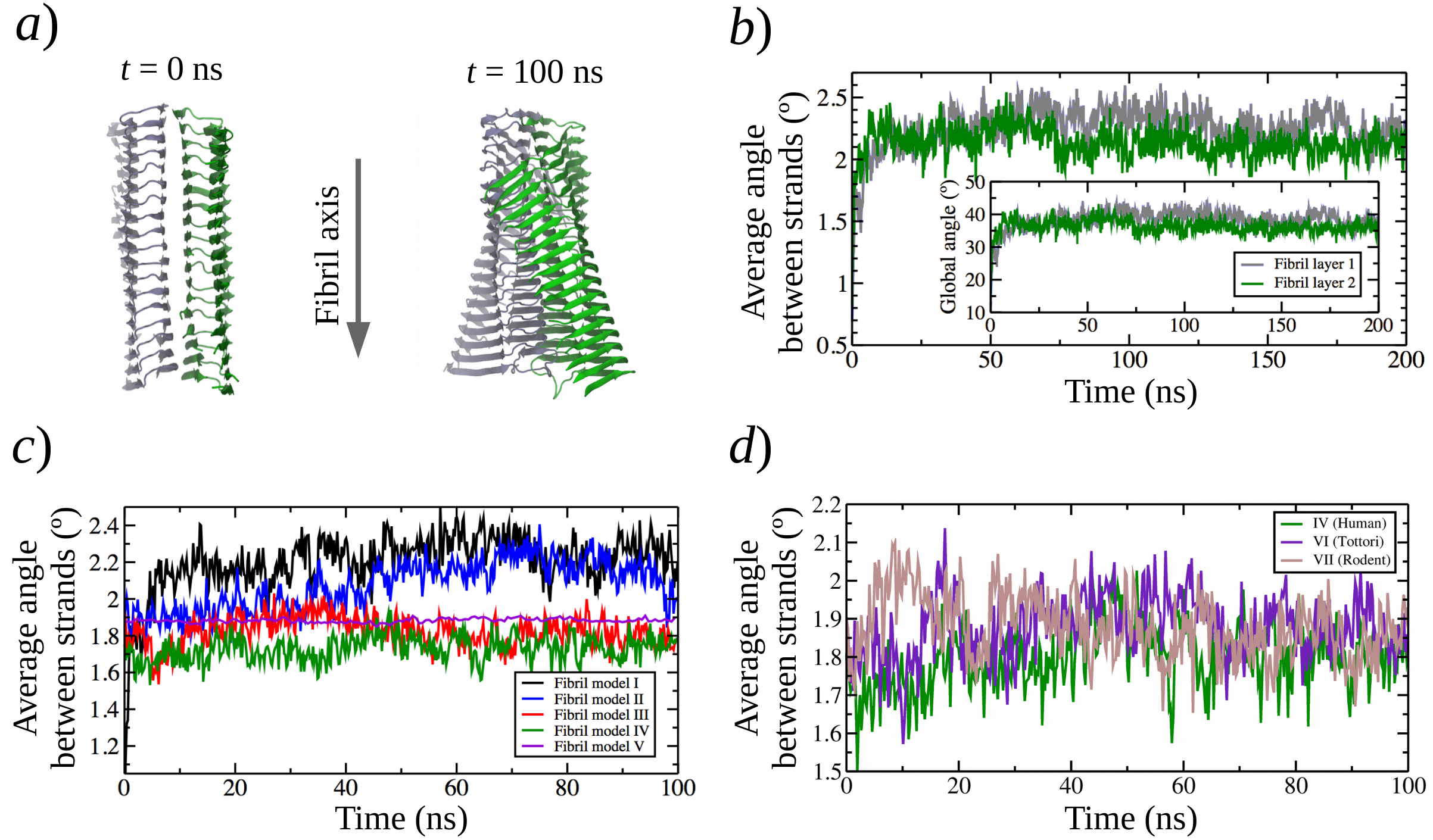


**Figure SI-3**. Twisting of the amyloid protofibril models. *a*) Initial and final side views of the structure of model I. *b*) Plot for the average angle between strands against the simulation time per protofibril layers of the model I. The inset of *b*) shows the plot of the global angle for the whole model against time, i.e., the relative angle between the first and the last strands of the fibril. *c*) Average angle between strands for models I-V. *d*) Average angle between fibril strands of models IV, VI, and VII. Note: The twist angle was measured as the dihedral angle taking four points: the first two corresponding to the alpha carbon of residues Ile41 and Ala30, located at the extremes of the inner β-sheet strands in the hydrophobic core of the models. Points 3 and 4 correspond to the alpha carbons of residues Ala30 and Ile41, respectively, from a different strand (either a neighboring or distant peptide chain). For distant chains the value of the dihedral angle was divided by the number of chains between the strands containing the points of measurement plus 1. For a given simulation time the twist angle was measured as the average of all pairs of strands in a model excluding two strands at each border of the aggregate, and in general both layers of the protofibril models were considered in the average. Finally, the time averages were calculated using the whole production simulation (100 ns) and sampling approximately every 50 ps.

**
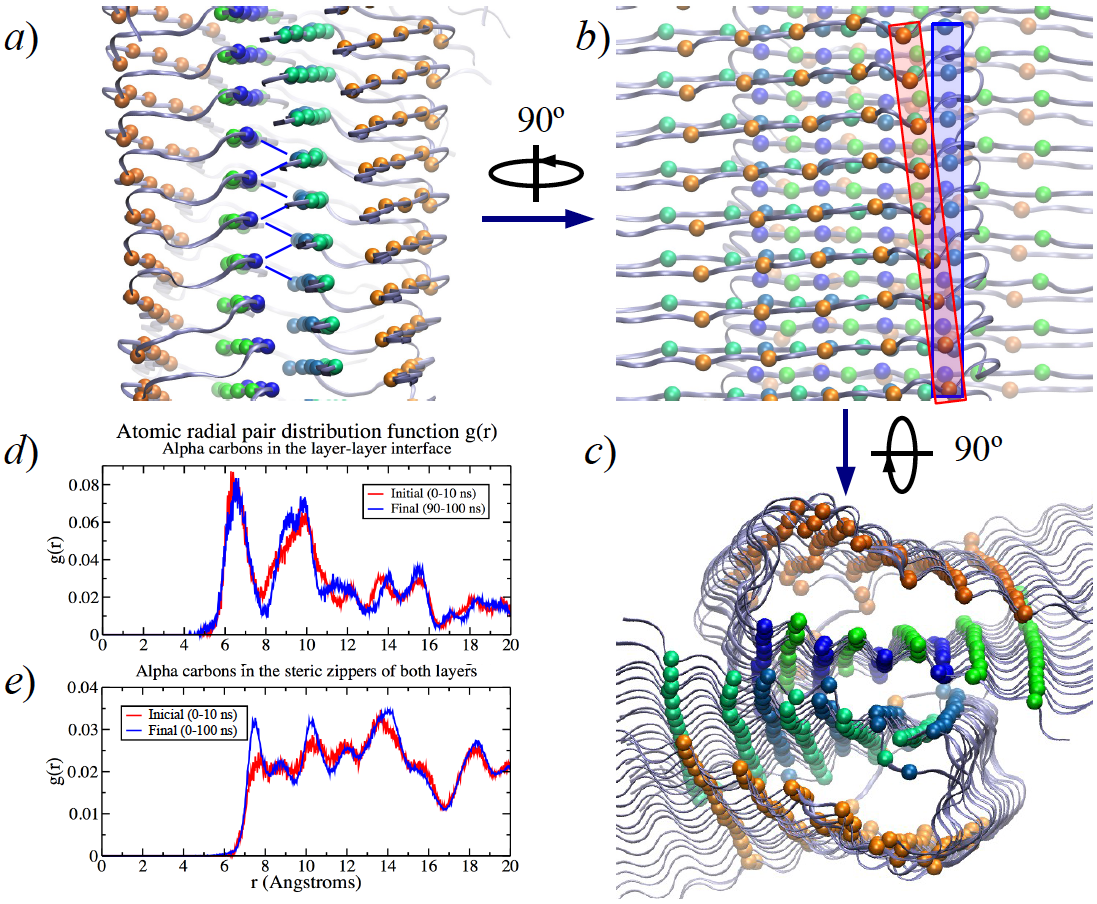
Figure SI-4**. *a*), *b*), and *c*) show the frontal, longitudinal, and transversal views of fibril model I highlighting the relative positions of the alpha-carbon atoms of the residues forming the steric zippers in the hydrophobic core. d) Distribution of distances between alpha-carbon atoms on the interlayer steric zipper in model I. *e*) Distribution of distances between alpha-carbon atoms in the intralayer steric zippers in model I.

**
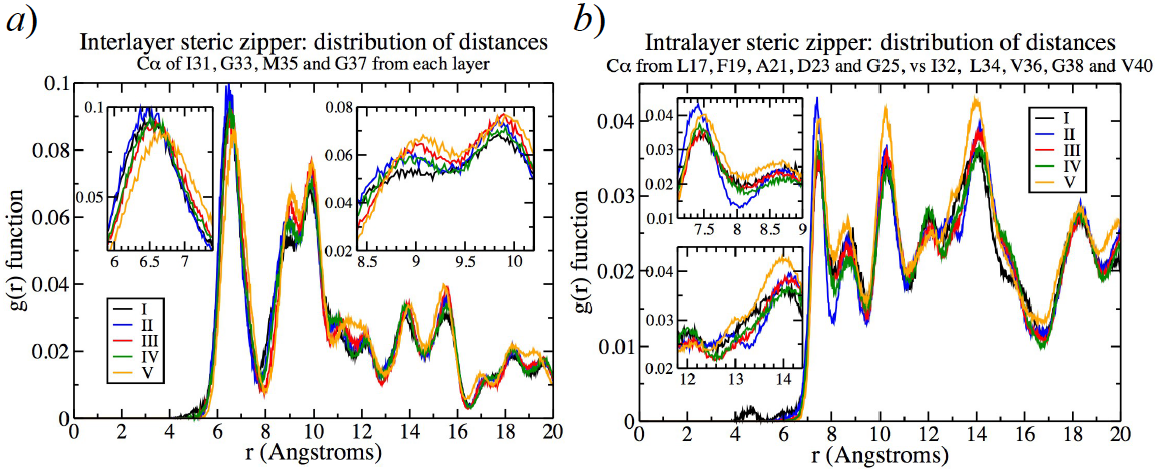
Figure SI-5.** Distribution of distances in the inter- (*a*) and intra-layer (*b*) steric zippers for the amyloid fibril models I-V, considering 100 ns of MD simulation.


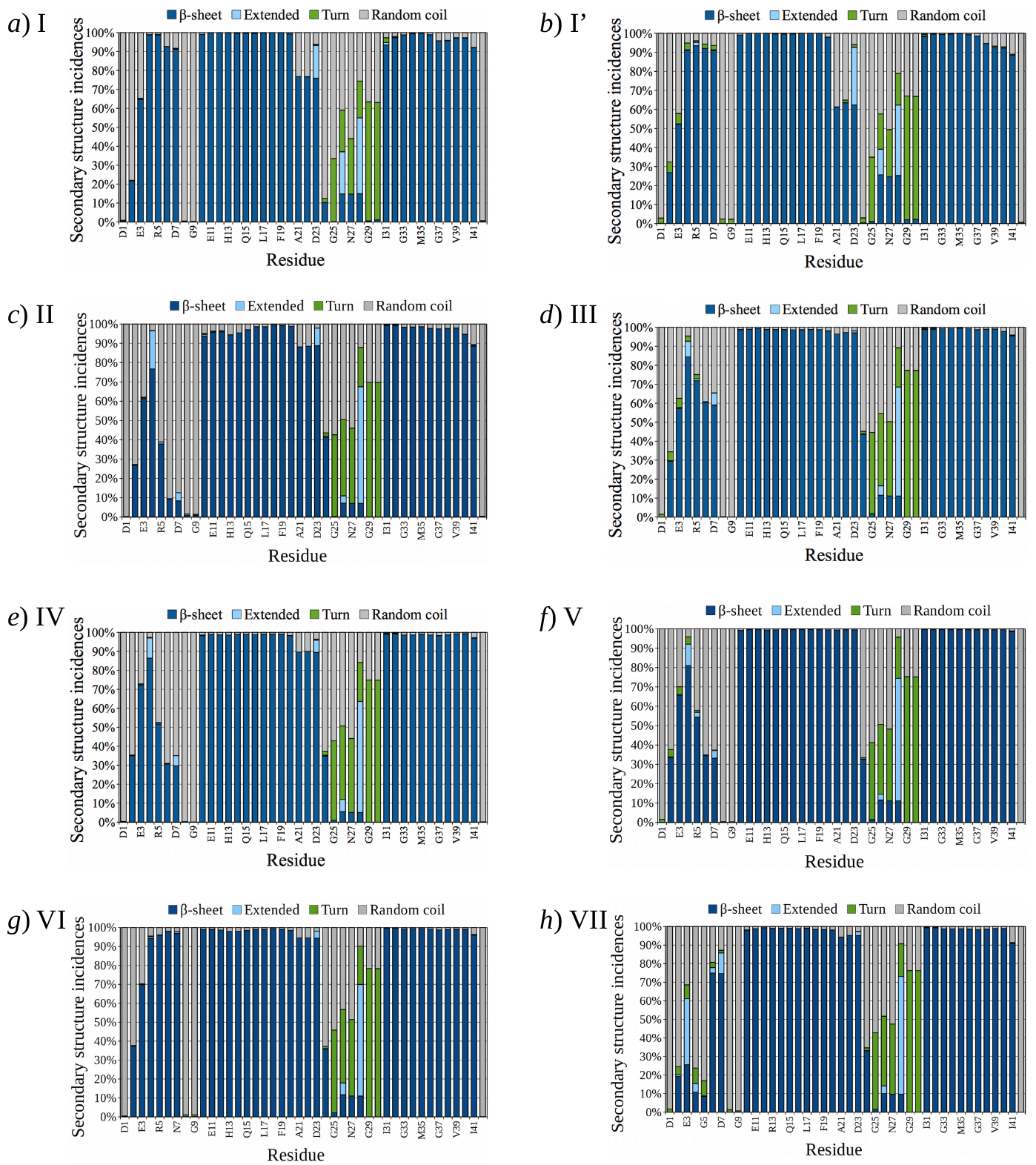


**Figure SI-6.** Incidence of protein secondary structure motifs per residue for the amyloid fibril models I-VII.

**
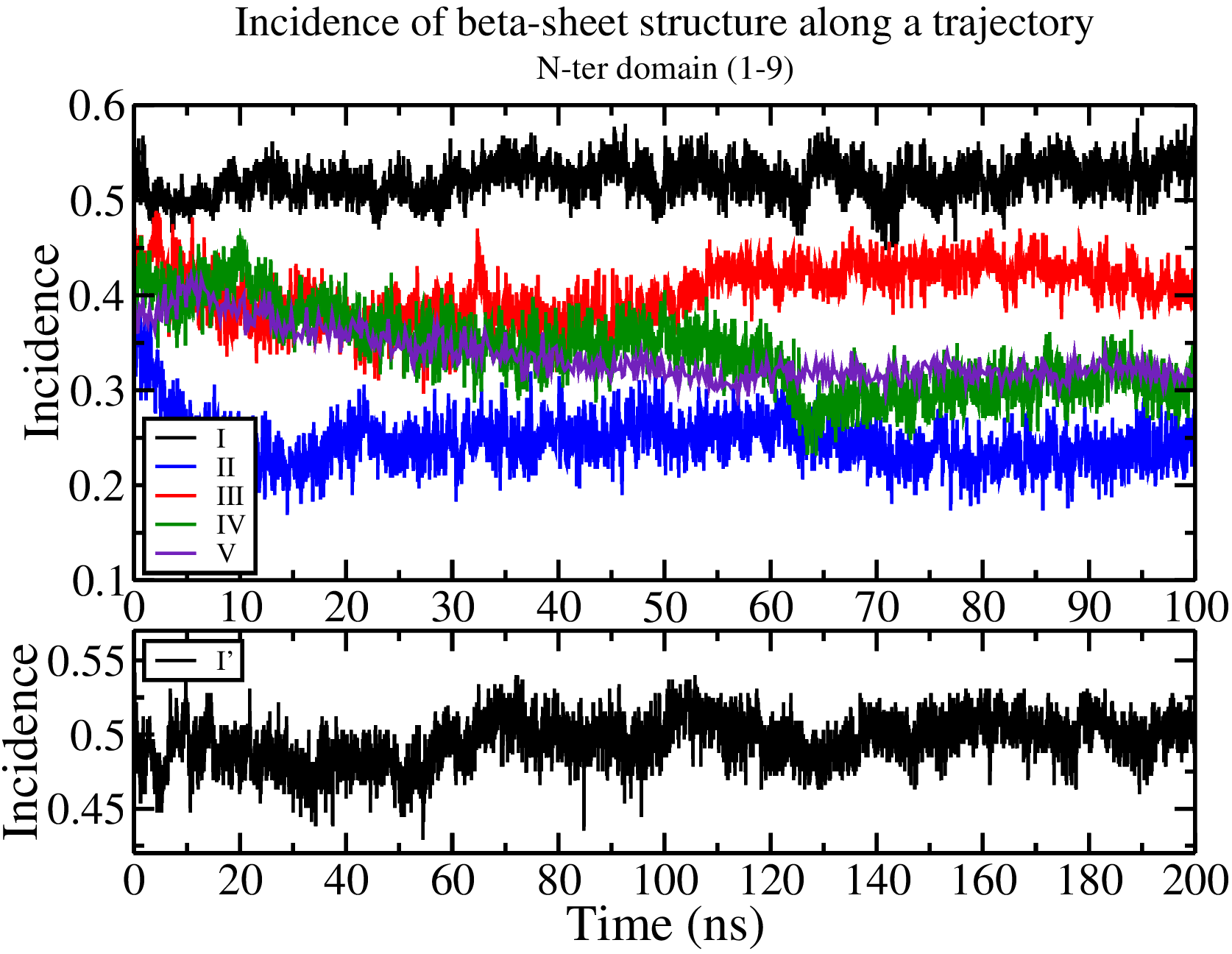
Figure SI-7.** Incidence of N-terminal (1-9) beta-sheet structure against time for protofibril models I-V. Model I’ represent an independent simulation (replica) of model I that was run for 200 ns.

**
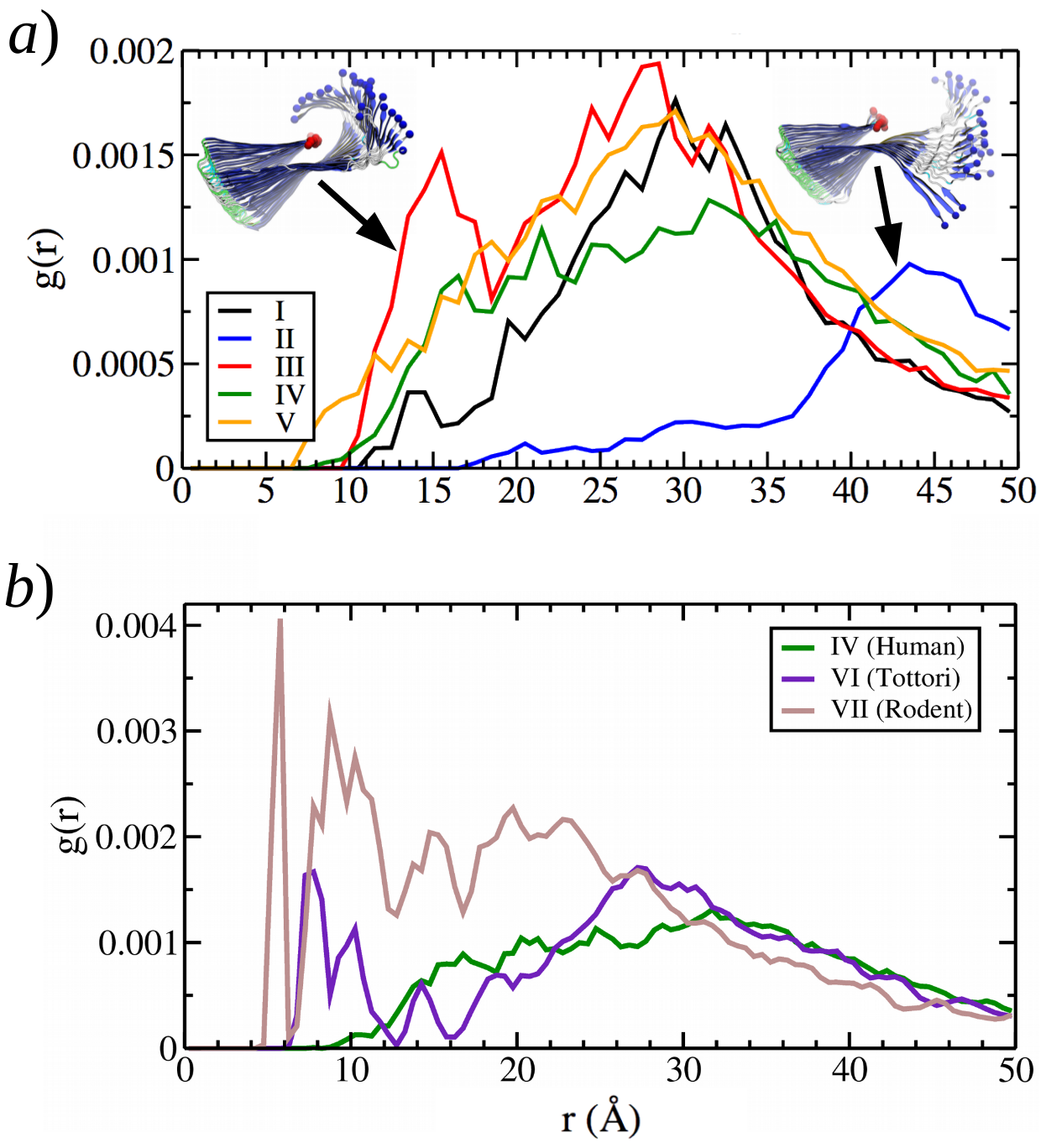
Figure SI-8.** Radial pair distribution function between C_α_ atoms from N-terminus (blue spheres) and C-terminus (red spheres) for the protofibril models during the last 5 ns of simulation. a) Models I-V. b) Models IV, VI, and VII.

**
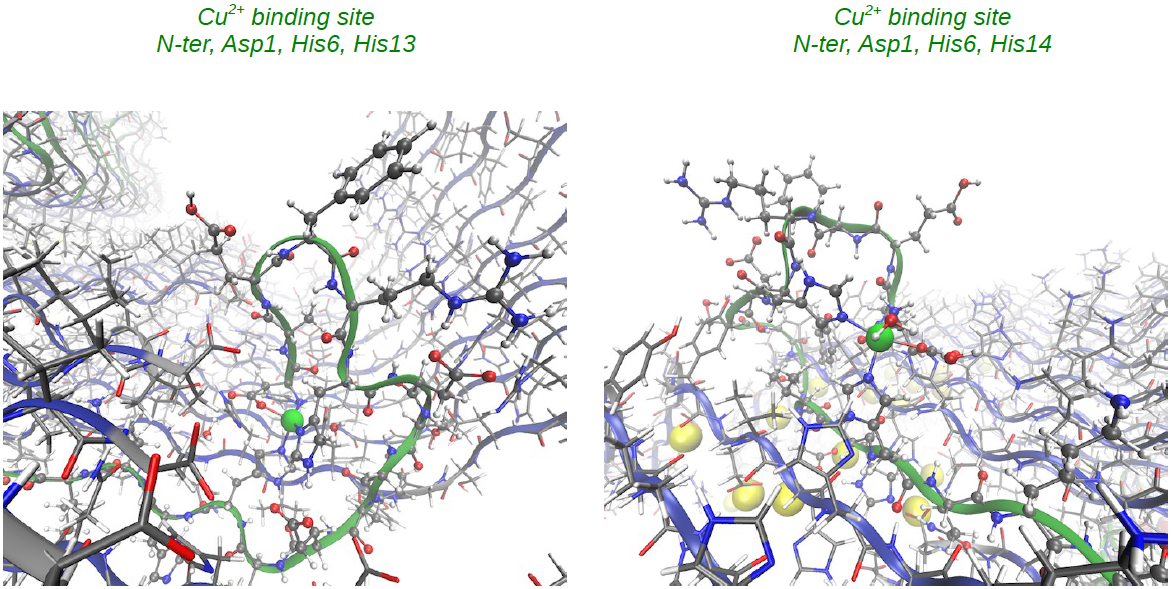
Figure SI-9**. Structure of Cu^2+^-A*β*_17_ complexes (green strands) superimposed to the structure of the amyloid fibril model I (blue strands). The complexes, reported in ref. 4, were optimized using all-electron DFT calculations starting from an extended conformation of the A*β*_17_ peptide as obtained from the model I after 50 ns of the MD simulations here presented. On the left, the binding site includes the His13, whereas, on the right, His14 is coordinating the copper ion (green sphere) on the opposite face of the N-terminal *β*-sheet. These figures show that the coordination of copper to the N-terminal domain is possible without affecting importantly the structure of C-ter residues from position 12.


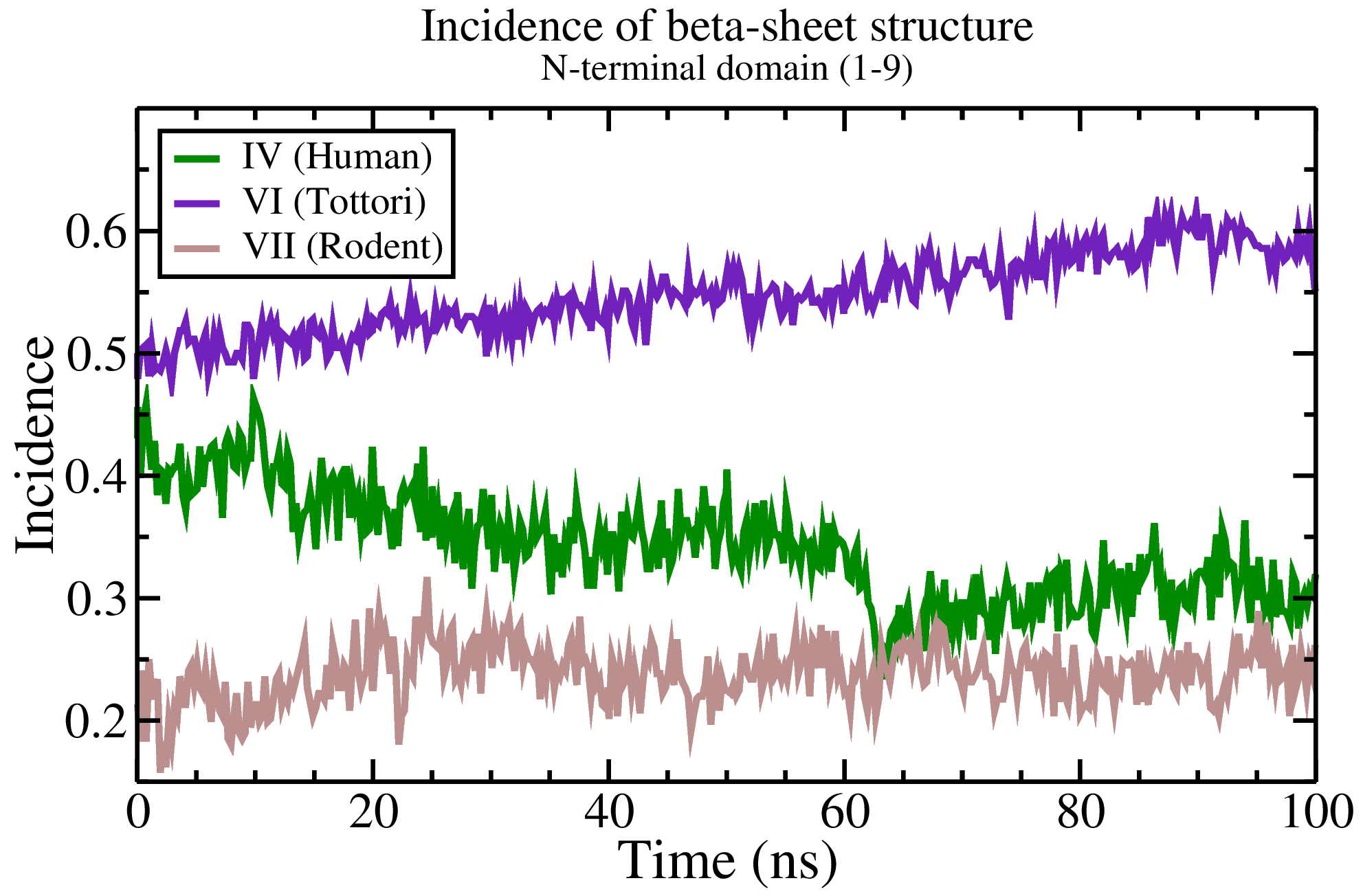
**Figure SI-10.** Incidence of N-terminal (1-9) beta-sheet structure against time for protofibril models IV, VI, and VII.

**
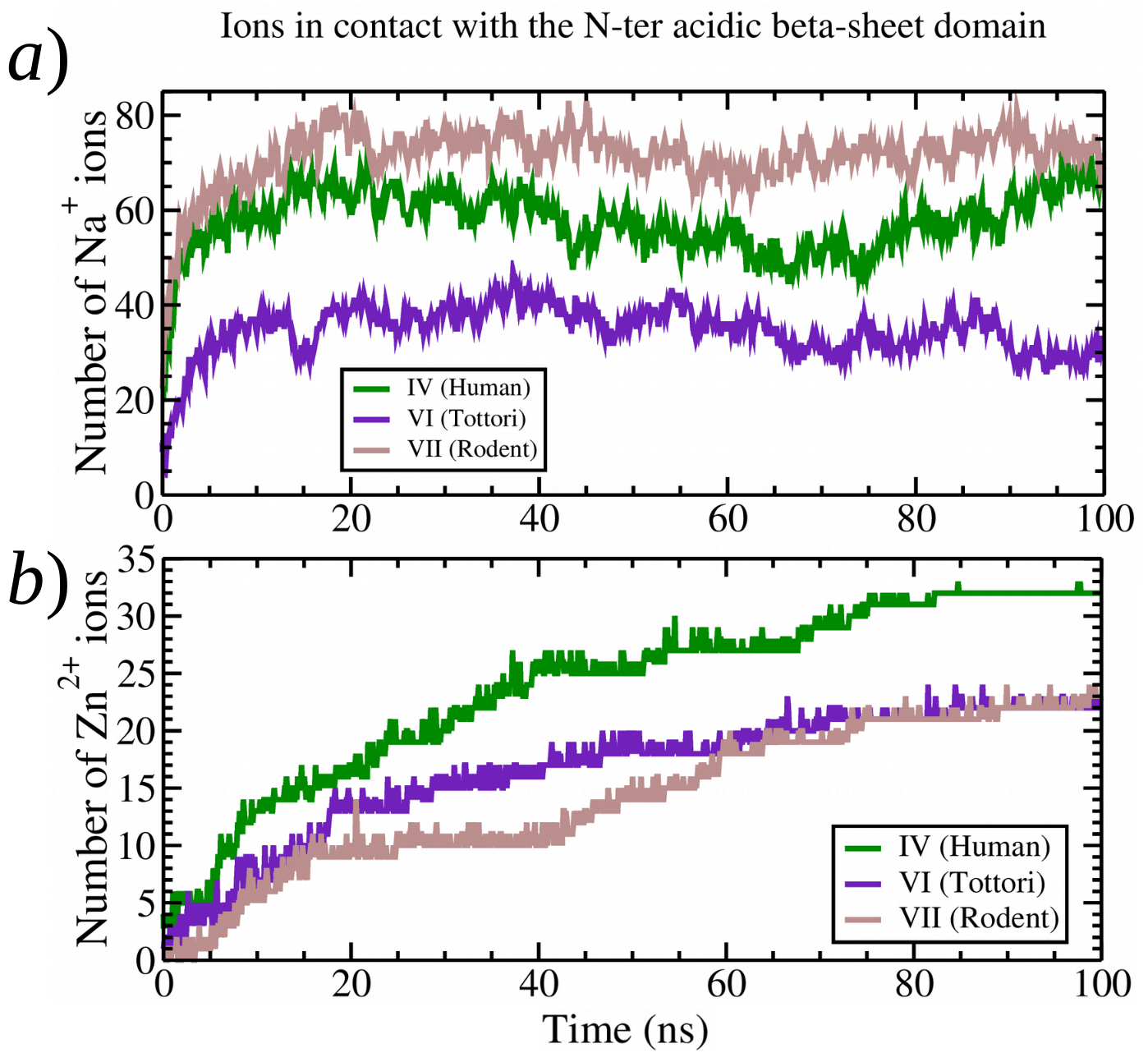
Figure SI-11.** Number of Na^+^ (*a*) and Zn^2+^ (*b*) cations in contact (within 3.5 Angstroms) with the N-terminal acidic *β*-sheet domain of amyloid protofibril models IV, VI, and VII.

**References**

1. Lührs, T.; Ritter, C.; Adrian, M.; Riek-Loher, D.; Bohrmann, B.; Döbeli, H.; Schubert, D.; Riek, R., 3D structure of Alzheimer's amyloid-beta(1-42) fibrils. *Proc. Natl. Acad. Sci. USA* **2005**, *102* (48), 17342-17347.
2. Humphrey, W.; Dalke, A.; Schulten, K., VMD: Visual molecular dynamics. *J. Mol. Graph.* **1996**, *14* (1), 33-38.
3. Petkova, A. T.; Yau, W. M.; Tycko, R., Experimental constraints on quaternary structure in Alzheimer's beta-amyloid fibrils. *Biochemistry* **2006**, *45* (2), 498-512.
4. Gomez-Castro, C. Z.; Vela, A.; Quintanar, L.; Grande-Aztatzi, R.; Mineva, T.; Goursot, A., Insights into the Oxygen-Based Ligand of the Low pH Component of the Cu^2+^-Amyloid-beta Complex. *J. Phys. Chem. B* **2014**, *118*, 10052-10064.
